# Identification of Partitivirus-like RdRPs in the *Brevipalpus yothersi* Genome Supports Viral-to-Arthropod Horizontal Gene Transfer

**DOI:** 10.3390/v18080892

**Published:** 2026-08-13

**Authors:** Bruno Afonso Corrêa, Thaís Medinilha Pancher, Denis Calandriello Calio, Aline Daniele Tassi, Laura Rossetto Pereira, Daniel Carrillo, Ricardo Harakava, Valdenice Moreira Novelli, Elliot Watanabe Kitajima, Pedro Luis Ramos-González, Juliana Freitas-Astúa, Daniel Gonzalez-Ibeas

**Affiliations:** 1 Instituto Biológico de São Paulo/IB, SAASP, São Paulo 04016-035, SP, Brazil; brunoafonso12@hotmail.com (B.A.C.); denis.calan@gmail.com (D.C.C.); laur.rossetto@hotmail.com (L.R.P.); ricardo.harakava@sp.gov.br (R.H.); plrg1970@gmail.com (P.L.R.-G.); juliana.astua@embrapa.br (J.F.-A.); 2 Agronomic Institute (IAC), Citrus Research Center ‘Sylvio Moreira’, Cordeirópolis 13492-442, SP, Brazil; thaismp@estudante.ufscar.br (T.M.P.); valdenice.novelli@sp.gov.br (V.M.N.); 3 Entomology and Nematology Department, University of Florida, Gainesville, FL 32611, USA; alinetassi@gmail.com (A.D.T.); dancar@ufl.edu (D.C.); 4 Escola Superior de Agricultura Luiz de Queiroz/ESALQ/USP, Universidade de São Paulo (USP), Piracicaba 13418-900, SP, Brazil; ewkitaji@usp.br; 5 Embrapa Cassava and Fruits, Cruz das Almas 44380-000, BA, Brazil

**Keywords:** acarology, false spider mite, endogenous viral element, transcriptome, protein structure, machine learning

## Abstract

RNA-dependent RNA polymerases (RdRPs) are essential enzymes involved in RNA virus replication and eukaryotic RNA silencing. They are generally absent in vertebrates but present in some invertebrate lineages, such as nematodes and certain arthropods. *Brevipalpus yothersi* is a phytophagous mite of agricultural relevance due to its role as a vector of plant-infecting viruses. We have identified two RdRPs on the genome of this mite species that, unexpectedly, are not of eukaryotic origin. Phylogenetic reconstruction and comparisons of 3D protein structures revealed similarity with viral RdRPs of the *Partitiviridae* family. Both RdRPs retain conserved catalytic motifs at the protein sequence level, and expression was confirmed by RNAseq and qPCR across mite developmental stages, with a peak during the nymphal stage. K-mer profiles showed similarity with mite endogenous genes, suggesting gene amelioration after the integration, or being derived from a viral donor already adapted to the mite. Our study also identified orthologs in other *Brevipalpus* species, but not in other Acari relatives, supporting that the horizontal gene transfer event is circumscribed to the *Brevipalpus* genus. These findings highlight an intriguing case of viral gene domestication in arthropods that might influence their developmental biology and the host–virus interaction.

## 1. Introduction

RNA-dependent RNA polymerases (RdRPs) catalyze the synthesis of RNA from an RNA template [1]. This activity is fundamental in several biological contexts, ranging from the replication of RNA viruses to the regulation of gene expression in eukaryotes [2]. Although the basic catalytic mechanism is conserved—nucleoside triphosphate polymerization guided by base-pair complementarity—RdRPs exhibit remarkable diversity in origin, structure, and function across plants, animals and viruses [3].

All RNA viruses, except retroviruses, rely on RdRP activity to replicate. Viral RdRPs are typically encoded in the viral genome and operate without a DNA stage, often within membrane-bound replication complexes in the cytoplasm of the host cell [4]. For example, the poliovirus (+ssRNA, Order *Picornavirales*) 3Dpol enzyme synthesizes both negative- and positive-sense RNA strands during infection. In negative-sense RNA viruses such as influenza (-ssRNA, order *Articulavirales)*, RdRPs not only replicate the genome but also produce subgenomic mRNAs for protein synthesis [5,6]. The λ3 polymerase (dsRNA, order *Reovirales*) operates within the viral core particle and synthesizes capped +RNA transcripts from each genomic dsRNA segment, which serve both as mRNAs for protein translation and as templates for synthesis of complementary negative strands during genome replication [7]. Viral RdRPs have evolved diverse strategies for initiation, proofreading, and processivity, reflecting the wide range of replication challenges in different viral families [8,9].

In plants, RdRPs play critical roles not only in viral defense but also in endogenous RNA silencing pathways, contributing to the control of transposable elements and gene regulation. Many plant genomes encode one or more RdRP genes, such as in *Arabidopsis thaliana* [10,11]. In animals, the distribution is patchier. In vertebrates, RNA interference (RNAi) exists but without the involvement of host-encoded RdRPs; instead, amplification of silencing signals is absent or minimal [12]. This absence may be compensated by other innate immune mechanisms, such as interferon responses, in higher animals [12,13]. Several invertebrate lineages, such as nematodes and some arthropods, possess functional RdRPs [14]. In *Caenorhabditis elegans*, for instance, RdRPs are central to RNA interference amplification, enabling robust and heritable gene silencing [15]. Arthropods present a particularly intriguing frontier in RdRP research. While most insects lack recognizable RdRP genes, certain chelicerates (e.g., ticks, mites, spiders) and crustaceans retain them [16,17]. Functional studies suggest that some arthropodal RdRPs may participate in antiviral RNA silencing, similar to plant systems, producing dsRNA intermediates that are diced into siRNAs [16].

Horizontal gene transfer (HGT) is the non-sexual movement of genetic information between genomes, an evolutionary process that contributes to genome diversity and adaptation [18,19]. Although abundant in prokaryotes, eukaryotic genomes harbor fewer elements coming from HGTs than bacteria and archaea [18]. In the case of viruses, host genes can be acquired during infection, incorporating them into their own genomes [20]. Conversely, viral sequences can integrate into eukaryotic genomes, creating endogenous viral elements (EVEs) that may persist across generations. These insertions can be neutral, deleterious, or even co-opted for beneficial roles, such as in placental development in mammals [21]. Although such insertions are more frequent for retroviruses and DNA viruses, cases from RNA viruses have also been proposed involving coat proteins and RdRPs [22,23,24,25,26]. In arthropods, RdRP HGTs might go further beyond viral boundaries. In *Strigamia maritima*, for example, phylogenetic analyses showed that one RdRP was more closely related *Neurospora crassa* than to other arthropodal sequences [27], and several insect RdRPs are more closely related to partitiviruses [26]. Within the Acari taxon, EVEs have also been reported in ticks, including RdRPs from totiviruses (dsRNA viruses) [28]. All those events are especially relevant in arthropods since they serve as vectors for RNA viruses (e.g., mosquitoes with flaviviruses, ticks with bunyaviruses, or mites with plant viruses), and the presence or absence of RdRPs could influence vector competence, virus persistence, virome composition and cross-species transmission.

*Brevipalpus* (Acari: Tenuipalpidae) mites include over 300 species that naturally infest 1000 plant species, causing economic losses in a large number of horticultural and ornamental plants through the transmission of viruses, including citrus, coffee and passion fruit [29,30]. *Brevipalpus yothersi* is responsible for the transmission of citrus leprosis virus C (CiLV-C, species *Cilevirus leprosis*), with particular relevance in Brazil and the rest of South America [31,32]. We used the current draft genome assembly [33] to screen for potential EVEs with similarity to RdRPs. Two candidate loci have been identified with strong molecular signatures of functionality. To our knowledge, this is the first report of viral RdRPs integrated into a mite genome. Implications for mite biology and host–virus interactions are discussed.

## 2. Materials and Methods

### 2.1. Identification of Endogenous Viral RdRPs

Protein sequences of viral and eukaryotic RdRps were downloaded from the RefSeq GenBank database with the Bio.Entrez package of Biopython (v1.85) [34] and from UniProt (12 September 2025) [35]. The *Brevipalpus yothersi* genome sequence was downloaded from the ftp of Genbank (accession GCA_003956705). The RdRP collection was blasted (tBLASTn, v2.12.0+) against the mite genome (E-value < 1 × 10^−10^, query coverage > 90%, identity > 90%). The mite transcriptome data were downloaded from the Sequence Read Archive (accession SRR7825313) with the SRA-tool-kit (https://github.com/ncbi/sra-tools, accessed on 10 July 2025). DNA reads were evaluated with FastQC (v0.12.1) [36], quality-filtered with Sickle (v1.33) [37], and *de novo* assembled with Trinity (v2.15.2) [38].

### 2.2. Protein Domain Analysis

Protein domain identification was performed with InterProScan (v5.75) [39], and a custom Python (v3.12.3) script was used for filtering the output in JSON format. The RdRp-scan [40] resource was explored with HMMER (v3.4) (http://hmmer.org/, accessed on 25 September 2025) (RdRp_HMM_profile.db.h3 profile) and DIAMOND (v2.1.13) (RdRp-scan_0.90.dmnd database) [41]. The RdRp motif database from RdRp-scan was used to analyze A, B, and C motifs. To search for functional sites and specific catalytic residues of the RdRP domains, a standalone version of the Reverse Position-Specific BLAST (RPS-BLAST) (v2.16.0+) [42] was used in combination with rpsbproc (version v0.5.0) [43] in two steps.

### 2.3. Phylogenetic Analysis

MAFFT (v7.505) [44] was used to perform multiple sequence alignments, RAxML Next Generation (v1.2.2-master) [45] for phylogenetic tree reconstruction, and ModelTest-NG (v0.1.7) [46] to select the best model. We set 500 bootstraps with autoMRE to check for convergence. Models BLOSUM62 + G4 and PMB + I were used for the viral/eukaryotic RdRPs and the viral RdRPs trees, respectively, to account for dataset size, taxonomic composition, and evolutionary depth. Protein sequences were obtained from the RdRp-scan repositories (CLUSTALO_0.4.fasta) [40]. SeqKit (v. 2.3.0) [47] was used for domain slicing. For tree plotting, a combination of iTOL (v7.2.2) [48] and Inkscape (v1.2) was used. Transfer Bootstrap Expectation [49] was used to assess branch support as it is especially suitable for large datasets.

### 2.4. Protein Structure Analysis

Protein 3D structures were downloaded from the RCSB Protein Data Bank (PDB) [50]. The AlphaFold 3 server (https://alphafoldserver.com, accessed on 20 August 2025) [51] was used to predict protein 3D structures not available in the PDB. For viral RdRPs, we chose the viruses Hubei partiti-like virus 42 (HPLV-42) (unclassified Riboviria); Citrus leprosis virus C (CiLV-C) (species *Cilevirus leprosis*, family *Kitaviridae*, phylum *Kitrinoviricota*); vesicular stomatitis Indiana virus (VSIV) (species *Vesiculovirus indiana,* family *Rhabdoviridae,* phylum *Negarnaviricota*); rabbit hemorrhagic disease virus (RHDV) (species *Lagovirus europaeus*, family *Caliciviridae*, phylum *Pisuviricota*). For eukaryotic polymerases, we chose three proteins from the mite *Tetranychus urticae*, a model organism in acarology, commonly known as two-spotted spider mite; *Dinothrombium tinctorium*, commonly known as red velvet mite; and *B. obovatus*. FoldSeek [52] was run online (https://search.foldseek.com/search, accessed on 31 January 2026).

### 2.5. Mite Rearing

Non-viruliferous *B. yothersi* mites were obtained from an isoline population maintained at the IAC-Citrus Research Center ‘Sylvio Moreira’, Cordeirópolis, SP, Brazil. Colonies were reared on jack bean leaves (*Canavalia ensiformis*) surrounded by moistened cotton at 25 ± 5 °C and 75 ± 5% relative humidity. To obtain synchronized developmental stages, adult females were transferred to prepared leaves for 48 h for oviposition and then removed. Eggs, larvae, nymphs, and adults were collected at defined time points after oviposition. The samples were flash frozen and stored at −80 °C until use. Leaves were renewed periodically to maintain colony stability and leaf turgor throughout the experiment. Viruliferous adult mites were obtained from an isoline population maintained at the Instituto Biológico, São Paulo, SP, Brazil. This population, known to transmit CiLV-C, was reared on sweet orange fruits (*Citrus sinensis*) under the same laboratory conditions described above.

### 2.6. Real-Time Quantitative PCR

Total RNA was extracted from pooled mite samples (eggs: *n* = 300; larvae: *n* = 400; nymphs: *n* = 300; adults: *n* = 300) following the manufacturer’s instructions for the miRNeasy Micro Kit (QIAGEN, Hilden, Germany), with three biological replicates per developmental stage. RNA concentration was measured using a NanoDrop ND-8000 microspectrophotometer (Thermo Scientific, Waltham, MA, USA), and RNA integrity was evaluated with an Agilent 2100 Bioanalyzer (Agilent, Santa Clara, CA, USA). First-strand cDNA synthesis was carried out using the GoScript™ RT Kit with random primers (Promega, Madison, WI, USA) (150 ng of total RNA, reaction volume of 20 µL).

qRT-PCR analyses were performed on a LightCycler 480 system (Roche, Basel, Switzerland) using the PowerTrack SYBR Green Master Mix (Applied Biosystems, Waltham, MA, USA), in a final volume of 10 µL with three technical replicates and fast cycling mode. Melting curve analysis was performed to verify amplification specificity. No-template controls were included to check for potential contamination, and no–reverse transcription controls (RT–) to assess genomic DNA presence. Amplification efficiency for each primer pair was determined using a standard curve generated from a five-point, 2-fold serial dilution of cDNA, and it resulted in >0.97 for the three genes. Relative gene expression levels were calculated using the comparative threshold cycle (2^−ΔΔCt^) method. The Elongation factor 1-alpha (EF1A) was used as endogenous reference gene for normalization. Primers (Table S1) were designed using Primer3 [53].

### 2.7. Analysis of the Mite RdRPs at the Nucleotide Level

Standalone ML model DeepVirFinder [54] was trained with the encode.py and training.py scripts using cutting lengths of input sequences 150, 300, 500, and 1000 with option -l. For training the model, we used as input the mite CDSs from gene models of the annotated genome [33,55], and the viral RdRP dataset used for EVEs identification. Q values from the dvf.py output were calculated with the R package qvalue [56]. CAI was calculated with the CodonAdaptationIndex of the Bio.SeqUtils module of Biopython [34], using as input orthologous genes of taxon Acari (6933) downloaded from the OrthoDB (v12) [57] with the URL-based interface of the API. Matches in *B. yothersi* were identified by BLAST with at least 50% coverage of the query sequence.

## 3. Results

### 3.1. The Genome of B. yothersi Encodes Two Putative Viral RdRPs

A collection of 45,844 viral RdRP protein sequences from public repositories was used for a tBLASTn search against the mite genome sequence. Two regions of about 1200 nt in length with sequence similarity to riboviruses were identified. Three hundred nt of upstream and downstream flanking sequences were added to the candidate regions for the ensuing analysis. In parallel, raw sequencing transcriptome data used in the genome project [33] were quality-filtered with Sickle and FastQC, resulting in 188,833,983 clean reads, which were used for a *de novo* transcriptome assembly with Trinity. The resulting 64,435 transcripts (grouped into 29,959 unique read clusters) were used for ORF identification and domain prediction. A total of 19 entries were identified with an RdRP-related Pfam [58] domain (Table S1), grouping into three read clusters (DN100, DN2153 and DN636) containing several putative genes and isoforms.

From cluster DN2153, the longest representative transcript TRINITY_DN2153_c0_g2_i1 had nearly-perfect sequence similarity with RNA1 of citrus leprosis virus C (CiLV-C, species *Cilevirus leprosis*) (accession MT554536, 99% identity, 100% coverage), and it was non-mappable to the mite genome, so we assumed a viral origin since CiLV-C viruliferous mites were used as a source for RNA and DNA isolation during the genome project [33]. Cluster DN636 comprised a complex array of 39 isoforms. The longest representative transcript TRINITY_DN636_c1_g1_i8 mapped to the mite genome to a single locus of scaffold QZCP01000123 (coordinates 174402-175841), overlapping with one of the previously identified RdRP-like areas of the genome. Out of 26 isoforms of cluster DN100, the longest representative transcript TRINITY_DN100_c0_g1_i88 was also mapped to a single locus on scaffold QZCP01000134 (coordinates 53096-54577), overlapping the second RdRP-like area. Both clusters DN636 and DN100 will hereafter be referred to as RdRP1 and RdRP2, respectively, as candidate mono-exonic EVEs in the *B. yothersi* genome.

### 3.2. RdRP1 and RdRP2 ORFs Show Complete Viral Protein Domains with Traits of Functionality

**Figure 1:**
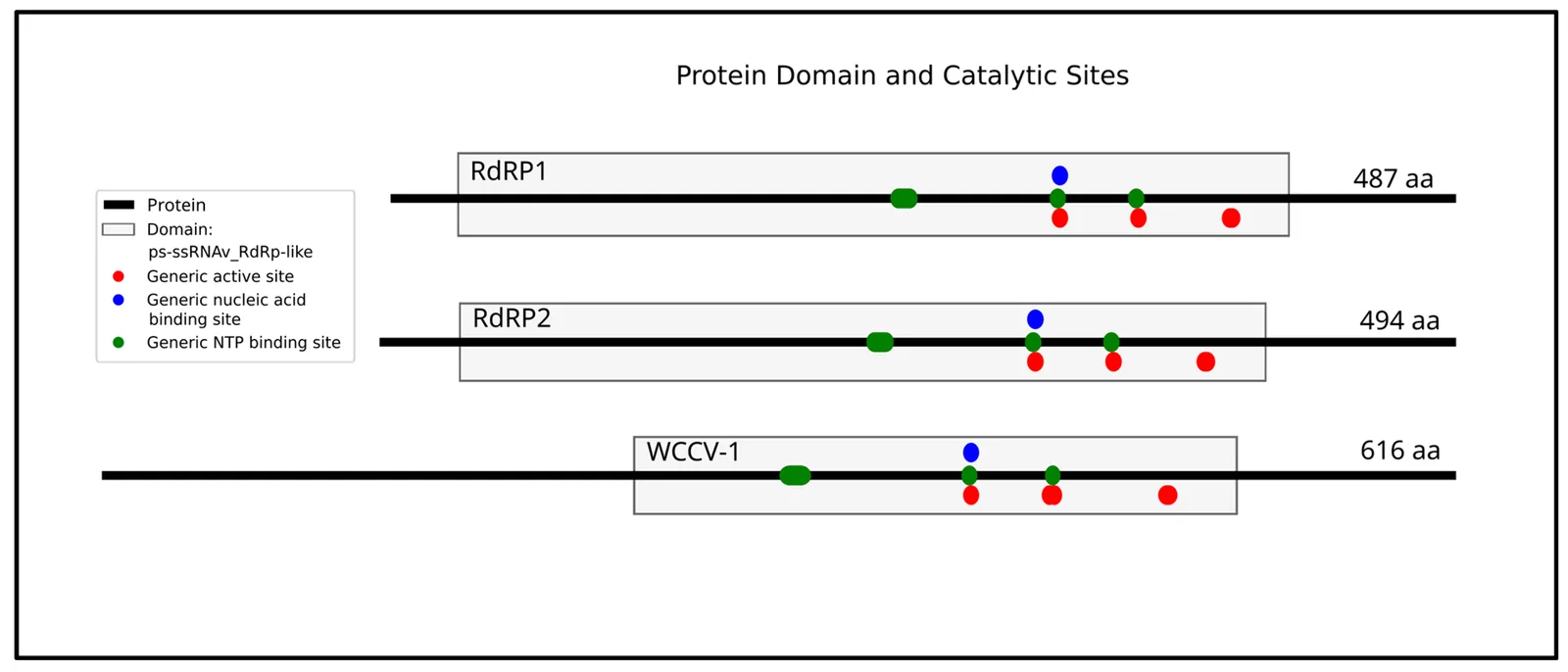
Protein domains and catalytic active sites identified by InterProScan and RPS-Blast on the deduced amino acid sequence from the ORF of RdRP1 and RdRP2 from *B. yothersi*, and the RdRP of white clover cryptic virus-1 (UniProtKB/Swiss-Prot accession AY705784).

Representative transcripts of both RdRPs contained a single complete Pfam domain PF00680, integrated into the InterPro IPR001205. Although many EVEs may be co-opted by hosts, the vast majority are assumed to accumulate mutations, frameshifts and genome rearrangements leading to disrupted, non-expressed and eventually non-functional elements [25,59]. Since the protein domain was complete and supported by RNAseq data, we searched for additional hallmarks of functionality. RPS-BLAST was used alongside the Conserved Domain Database (CDD) to confirm the conservation of catalytic residues that make the polymerase active, by comparison with other viral RdRPs. Both proteins were found within the CDD cluster cl40470. Several active sites related to nucleic acid binding were present (Figure 1), with a topology similar to the RdRP from the white clover cryptic virus 1 (WCCV-1, family *Partitiviridae*, order *Durnavirales*).

Both mite RdRP candidates were also functionally annotated with RdRp-scan using both sequence-based (DIAMOND) and Hidden Markov Models (HMMER) methods. Mite RdRPs showed high similarity with the RdRP of *Durnavirales* (e.g., Hubei partiti-like virus 42, accession KX884166, E-values < 2.28 × 10^−119^). The RdRp-scan resource contains a collection of motifs, A, B and C, from different taxa of viruses that are broadly conserved in RdRPs and are located in the palm subdomain, responsible for the catalysis [1]. All three motifs (Motif A (DFK×FD), B (SGS×FT×L×G), and C (DD)) were identified in the mite-derived RdRPs (Text S1).

### 3.3. Viral Origin of Mite Endogenous RdRPs Is Supported by Phylogenetic Inference

We used a phylogenetic approach to confirm the origin of the two identified mite RdRPs, including a selected set of protein sequences of either viral or eukaryotic origin. For the viral sequences, we used seven entries of the main viral branches following the scheme proposed by Wolf and others [60]. For branch 1, corresponding to phylum *Lenarviricota,* we chose ourmia melon virus (OuMV) (accession YP_002019757); for branch 2, corresponding to phylum *Pisuviricota,* we chose white clover cryptic virus 1 (WCCV-1) (accession AY705784) from the order *Durnavirales* and Solenopsis invicta virus 3 (SINV-3) (accession YP_002790880) from the order *Picornavirales*; for branch 3, corresponding to phylum *Kitrinoviricota*, we chose citrus leprosis virus C (CiLV-C) (accession DQ352194); for branch 4, corresponding to phylum *Duplornaviricota*, we chose rice dwarf virus (RDV) (accession BAA14222); for branch 5, corresponding to *Negarnaviricota*, we chose vesicular stomatitis Indiana virus (VSIV) (accession NP_041716), and rice stripe virus (RSV) (accession BAA06677). For the eukaryotic RdRPs, we selected one from the mite *Tetranychus urticae* (accession CAEY01000813) and three from other well-studied RdRPs in the literature, corresponding to *Arabidopsis thaliana* (accession NM_101348) [61], *Caenorhabditis elegans* (accession AF159144) [62] and *Neurospora crassa* (accession AJ133528) [63].

**Figure 2:**
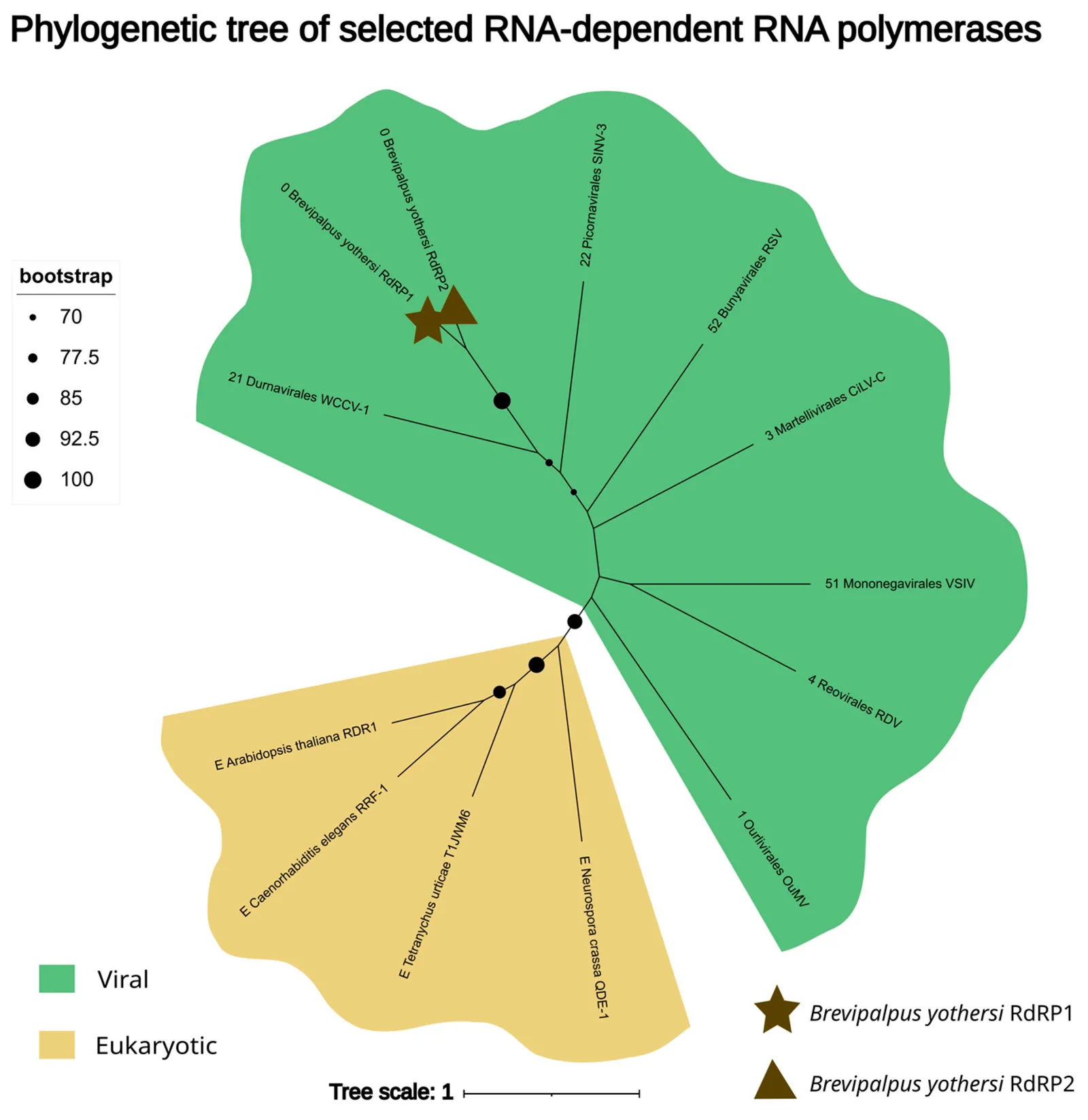
Phylogenetic tree of eukaryotic and viral RdRPs. Unrooted phylogenetic tree of viral (green) and eukaryotic (yellow) RdRPs, along with RdRP1 and RdRP2 from *Brevipalpus yothersi*. The numbers/letter represent the following: 0: *Brevipalpus yothersi*; 1: *Lenarviricota*; 2: *Pisuviricota*; 3: *Kitrinoviricota*; 4: *Duplornaviricota*; 5: *Negarnaviricota*; E: eukaryotic.

To avoid interference with non-RdRP sequences in multi-domain polymerases (e.g., from CiLV-C), we used only the RdRP domain region of the proteins predicted by CDD for the alignment with MAFFT. Because only a few sequences were used as input, the L-INS-i method was selected. RaXML-NG was used to infer the tree with the model BLOSUM62 + G4, due to a small mixed viral–eukaryotic dataset with proteins showing a medium to high level of divergence, and a gamma model to consider different substitution rates among sites, as suggested by ModelTest-NG. In the tree, eukaryotic and viral RdRPs were clearly grouped into different clusters (Figure 2), and *B. yothersi* proteins grouped with viral counterparts close to *Durnavirales*. Branch support was provided by bootstrapping.

To refine the phylogenetic allocation, a broader range of viral RdRPs was used from the RdRp-scan repository. For consistency, the RdRP domain regions were extracted from the protein sequences, aligned with MAFFT, and fed into RaXML-NG, using the model PMB + I, as suggested by ModelTest-NG. PMB is commonly used in protein analyses because it attempts to capture real amino acid substitution patterns in conserved regions; +I was intended to consider a proportion of invariant sites that never change throughout evolution (~2.17% of the sites were estimated to be invariant according to ModelTest-NG) [64]. Both RdRPs of *B. yothersi* were positioned within the clade of *Durnavirales* (Figure S1).

### 3.4. Expression of RdRP1 and RdRP2 Genes Display Distinct Patterns Across Developmental Stages

**Figure 3:**
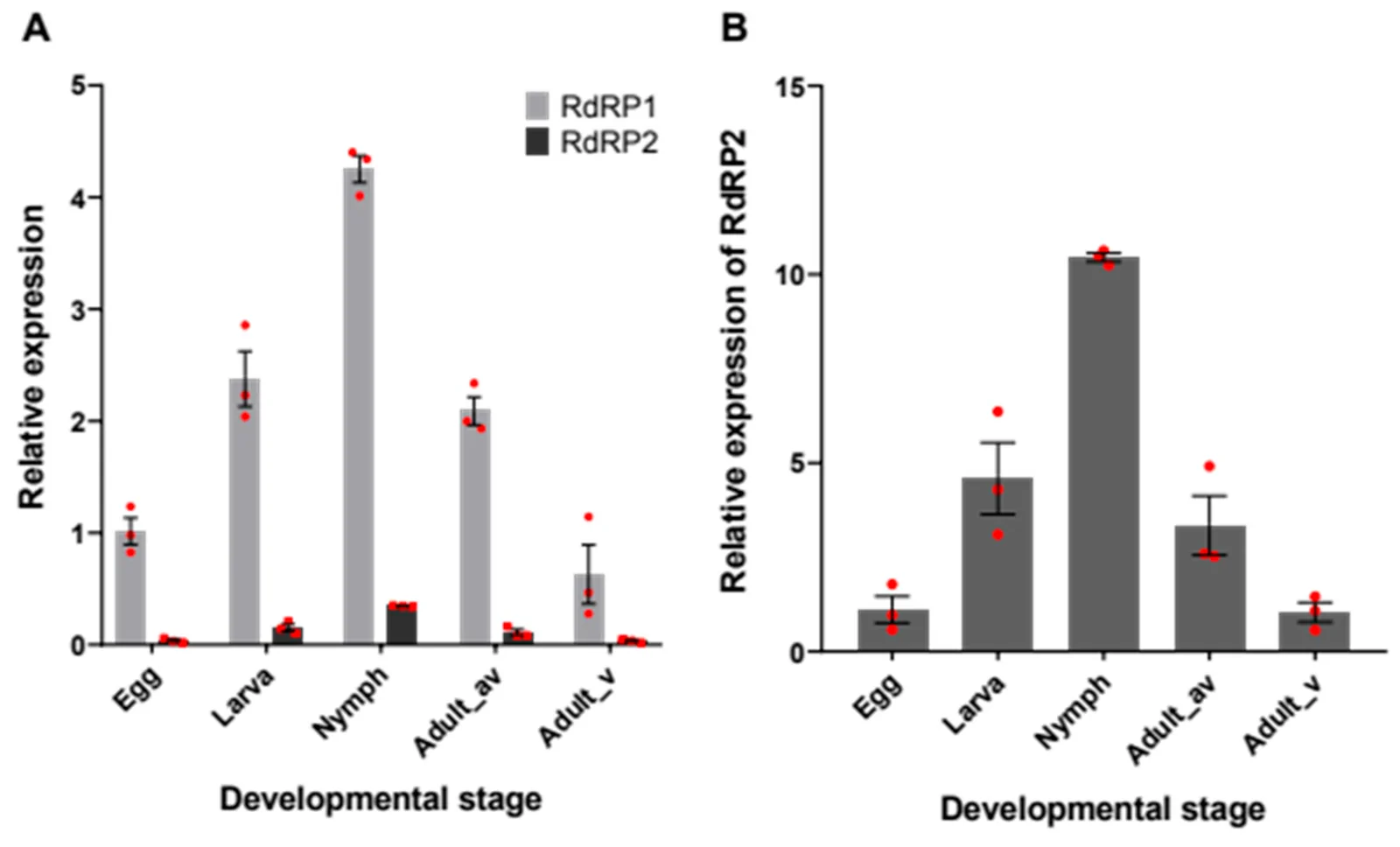
Expression patterns of RdRP genes across the four developmental stages of *Brevipalpus yothersi*. (**A**) Relative expression levels of RdRP1. (**B**) Relative expression levels of RdRP2. The calibrator for the 2^−ΔΔCt^ analysis was defined as the mean expression value of the egg stage, calculated separately for each RdRP gene. Bars represent mean relative expression values from three biological replicates (red dots), each consisting of 100 pooled individuals. Error bars indicate the ± standard error of the mean (SEM).

To assess the expression patterns of the two RdRP genes, a quantitative reverse-transcription PCR (qRT-PCR) assay was performed across the four developmental stages of the mite. All primer pairs exhibited comparable amplification performance, with amplification factors ranging from 1.69 to 1.71 (Table S2) and produced a single peak in melting curve analyses, indicating specific amplification. RNA accumulation analysis revealed similar stage-specific expression patterns for both RdRP genes, although expression was clearly higher for RdRP1 than for RdRP2 (Figure 3), in agreement with the RNA-seq quantification. Both genes showed a moderate increase from egg to a peak at the nymph stage, followed by a decline (Figure 3). RNA accumulation was also measured in CiLV-C-viruliferous mites, and normalized data showed a down-regulation relative to aviruliferous mites (Figure 3).

### 3.5. Orthologs of RdRP1 and RdRP2 Are Identified in Other Brevipalpus ssp.

A publicly available draft genome sequence was recently released for *B. obovatus* [65]. Using the same methodology, we identified four loci with similar rates of coverage and sequence identity to the RdRPs from *B. yothersi*. An all-vs-all matrix comparison of the deduced amino acid sequences of those genes with *B. yothersi* is provided in Figure S2. The four loci overlapped with four gene models functionally annotated as “uncharacterized proteins” by the NCBI metadata, and we confirmed that their expression was supported by the RNAseq data linked to the *B. obovatus* genome project.

The identification of RdRP orthologs was extended to a transcriptome data set of several *Brevipalpus* species part of the *Brevipalpus* virome project (Table S3) [66]. At least two copies were found on different species with proper coverage, sequence identity, and presence of the Pfam domain, including three transcripts from *B. chilensis*, two from *B. papayensis*, three from *B. californicus,* and two from *B. obovatus* (Table S3)*. B. cuneatus* was the only species in which no RdRP orthologs could be found. We also identified RdRP1 and RdRP2 in *B. yothersi*, providing additional RNAseq support from this new transcriptome data. A phylogenetic tree constructed with all identified RdRP protein sequences resulted in two clusters of RdRP1- and RdRP2-related sequences, suggesting orthologous relationships, and within each cluster, the sequences mirrored the phylogenetic hierarchy of the *Brevipalpus* genus [67]. This pattern is consistent with an integration event that occurred in a common ancestor and was followed by subsequent duplications, rather than resulting from independent HGTs (Figure S3). The main discrepancy came from one RdRP identified in *B. chilensis* (brchi.Viral, Figure S3). While brchi.RdRP1 and brchi.RdRP2 clustered coherently with the two putative orthogroups, brchi.Viral clustered close to the RdRP of HPLV-42 used as an outgroup. In the absence of a reference genome sequence for *B. chilensis*, drawing conclusions becomes more difficult, but this result suggests only two high-quality RdRPs in *B. chilensis* (as seen in *B. yothersi*), and brchi.Viral being a viral sequence from the virome. In turn, brchi.Viral shows sequence similarity with the Vespula vulgaris Partiti-like virus 1 RdRP (accession QZZ63312.1), identified as a persistent infection in the wasp virome in different developmental stages [68]. This RdRP was identified (identity > 98%, with complete Pfam domain) in six independent virome samples of *B. chilensis* collected from four different plant species in Chile (*Ligustrum, Vitis, Cestrum, and Vinca*). The other discrepancy came from transcript TRINITY_DN18007_c0_g1_i1 from *B. obovatus* (brobo.Viral, Figure S3), which was phylogenetically close to RdRP1 from *B. yothersi*. However, it did not map to the *B. obovatus* genome, consistent with being part of the virome of *B. obovatus* but not a genomic RdRP. Indeed, co-location within a cluster with the genomic RdRPs of *B. yothersi* would also break the orthologous relationships identified for the other *B. obovatus* genomic RdRPs, since *B. obovatus* and *B. yothersi* belong to separate phylogenetic groups [67], further supporting virome origin. This result suggests that brchi.Viral and brobo.Viral are good candidates to be the viral donor (or at least close relatives) of the HGT event. Neither of the two had a representative transcript in the *B. yothersi* virome. We also explored potential orthologs across the genome of 21 Acari (mites and ticks) species for which high-quality genome sequences are available in GenBank, but no hits were retrieved.

### 3.6. Viral Origin of the Mite RdRPs Is Also Supported by Computational Analysis of Their Protein 3D Structures

It is widely acknowledged that, in general, protein structure is more conserved than the primary sequence across evolution [69,70,71], which is also true in the particular case of viral RdRPs [72]. Thus, although sequence comparisons have for decades been the most common way to assess phylogenetic signals, mostly due to their affordability and the scarcity of available 3D structures so far, remote homology can be detected to a better extent using structural comparisons rather than protein sequences. The advent of machine learning approaches to infer 3D structures is prompting a methodological shift [69,73]. To further confirm the viral origin of the two identified RdRPs of *B. yothersi*, we predicted their 3D structure with AlphaFold 3 and compared them with a selected set of RdRPs of either viral or eukaryotic origin based on the results from the phylogenetic analyses, or availability of well-characterized structural data (Table 1, and see Section 2).

For the structure comparisons, we used the Pairwise Structure Alignment Tool from the Research Collaboratory for Structural Bioinformatics Protein Data Bank (RCSB-PDB). The proteins of rabbit hemorrhagic disease virus (RHDV) and vesicular stomatitis Indiana virus (VSIV) were available at the PDB; the others were predicted using AlphaFold 3. Only the domain region from the whole protein sequence predicted by InterProScan was used. We chose TM-align, a rigid-body structure aligner, to find the best structural match between proteins, which is sequence-independent, and more sensitive to the global topology than to local structural variations [74]. Root mean square deviation (RMSD), TM-score, identity and aligned residues were used for quality assessment. RMSD is a measure of the mean distance of Cα of residues, showing whether the proteins adopt similar conformations. The smaller the values, the more similar the conformations, but this value tends to grow for big proteins, so TM-score can be a better metric in those cases, as it normalizes the values using the smaller protein length. Identity refers to the percentage of conserved residues compared to totally aligned ones. Aligned residues are the number of residues of the proteins that were aligned. Since domain sizes varied substantially among proteins, the number of aligned residues was normalized by the mean number of modeled residues.

**Figure 4:**
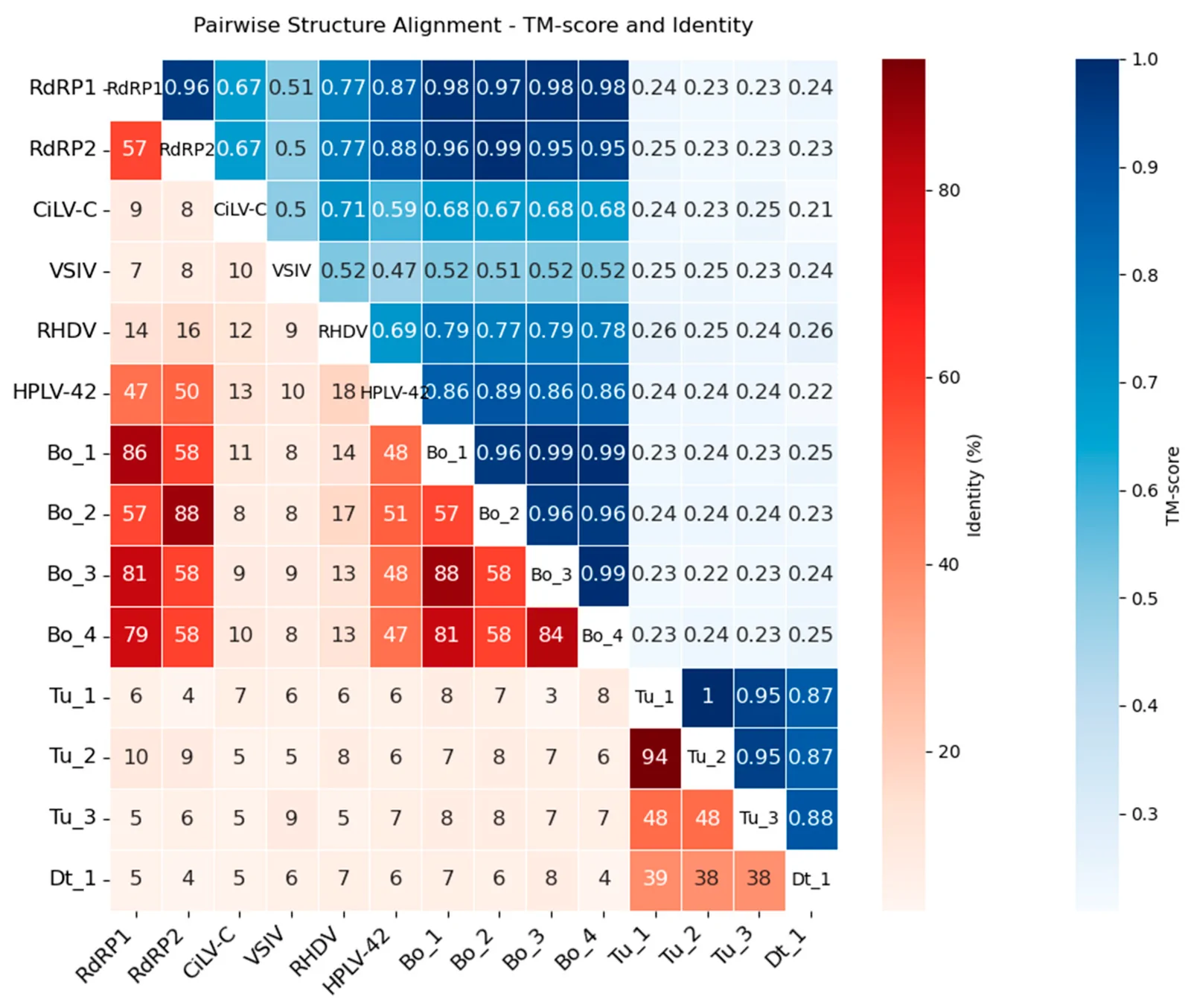
TM-score and identity from a pairwise structure alignment of selected RdRPs. Dual triangular matrix representing TM-score in the upper half (blue) and identity in the lower half (red). For information about the proteins represented, see Table 1.

**Figure 5:**
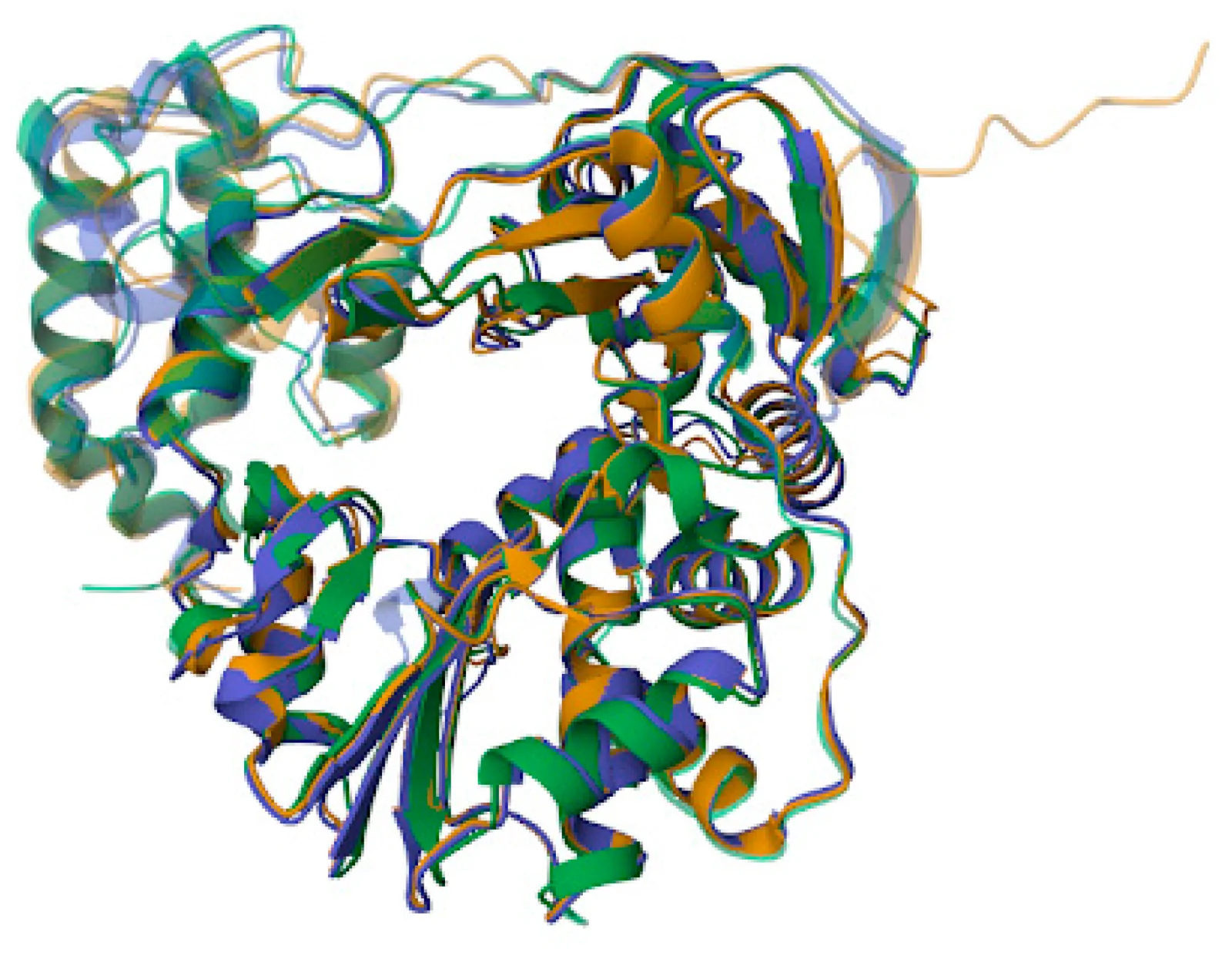
Hubei partiti-like virus 42 (green) 3D structure aligned with *B. yothersi* RdRP1 (orange) and RdRP2 (purple). Transparent areas represent residues not overlapping with the RdRP domain predicted by Pfam.

*B. yothersi* RdRPs clearly showed higher TM-scores and identities with viral proteins than with their eukaryotic counterparts (Figure 4), underscoring their viral origin. The highest similarity was achieved for the dsRNA virus Hubei partiti-like virus 42 (HPLV-42). Both parameters were also markedly higher when the RdRPs from *B. obovatus* and *B. yothersi* were compared, which underpins orthologous inheritance rather than independent HGT events. Interestingly, the differences in primary amino acid sequence identity of the proteins among RdRP members of *B. obovatus* and *B. yothersi* were dampened in the TM-scores, highlighting how evolutionary variations in protein sequence do not translate into major variations in the global protein structure. A similar observation can be made from the comparison within the eukaryotic RdRPs of *T. urticae* (Figure 4). Similar conclusions were drawn from RMSD and aligned residues (Figure S4). A representation of overlapping 3D structures of RdRP1, RdRP2 and HPLV-42-derived RdRP is shown in Figure 5.

A more unsupervised search was conducted with both *B. yothersi* RdRPs against the FoldSeek databases (AlphaFold/Proteome, AlphaFold/Swiss-Prot, AlphaFold/UniProt50, PDB100, BFMD, BFVD, CATH50, GMGCL, Mgnify-ESM30). Results were consistent with our previous findings, identifying several viruses from the partiti-like group and the order *Durnavirales* (Table S4).

### 3.7. Nucleotide Sequence of Both RdRPS Mirror the K-Mer Profiles of the Mite Genome

The K-mer content of DNA sequences allows for the identification of EVEs of either DNA or RNA origin by detecting genomic signatures that differ between eukaryotic hosts and their source viruses [24]. Once the two putative virus-derived mite RdRPs of *B. yothersi* were characterized at the protein level, we trained the machine-learning (ML) algorithm DeepVirFinder (DVF) [54] to classify them as viral or eukaryotic at the nucleotide level. To train the model, we used as input the mite CDSs from gene models of the annotated genome [33,55], and the viral RdRPs from NCBI used for EVEs identification. Surprisingly, both mite RdRPs were classified by the trained model as eukaryotic mite sequences, instead of viral (Table S5A). Since genomic areas have different evolutionary dynamics at the nucleotide and protein levels (e.g., synonymous/non-synonymous mutation rates, or codon usage adaptation bias) [75], we hypothesized that, assuming a viral origin, the k-mer content of the mite RdRPs may have shifted towards the mite’s profile through “sequence amelioration” as a consequence of endogenization [76]. Among the multiple factors that may drive this k-mer transition, we focus on the analysis of codon usage amelioration since it greatly impacts the success of HGTs in bacteria [77,78], and there is a relationship between k-mer profiles and compositional biases in nucleotide sequences linked directly or indirectly to codon usage [79,80,81,82].

**Figure 6:**
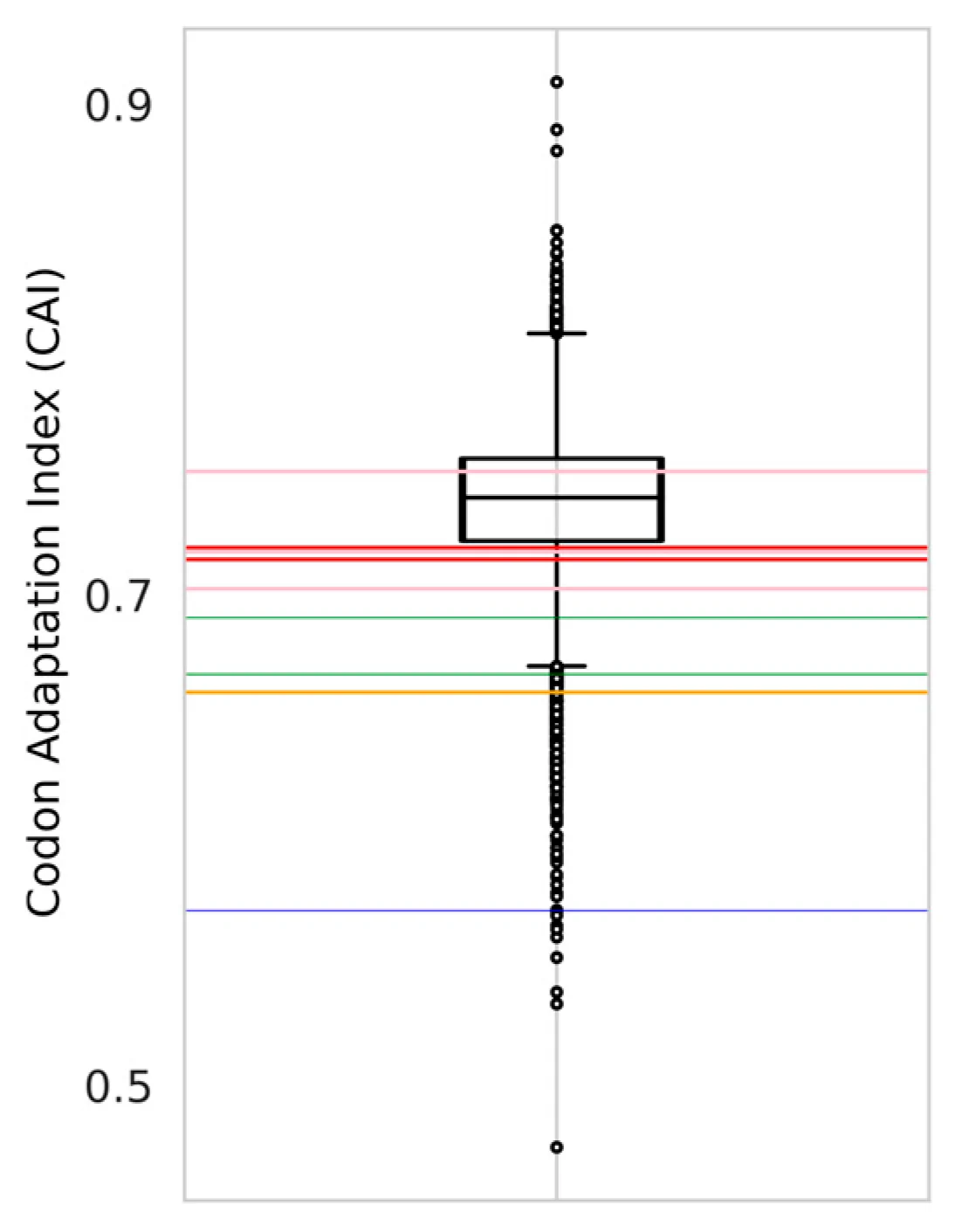
Codon adaptation index (CAI) calculations of *B. yothersi* protein-coding gene annotations (boxplot), *B. yothersi* genomic RdRPs (red lines), viral RdRPs of HPLV-42 and WCCV-1 (*Durnavirales*, green lines), and viral RdRPs derived from viruses known to replicate in the mite, including ClCSV (*dichoravirus*, blue line), CiLV-C (*Cilevirus*, orange line), and viral RdRPs identified in the virome data (pink lines). Black dots represent outliers.

We used the Codon Adaptation Index (CAI) [83] to measure how strongly the codon usage of the mite and other viral RdRPs match the preferred codons of endogenous mite genes. The mite RdRPs showed CAI values (0.71 and 0.72) below the average of mite genes (0.75), but slightly higher than those of the viral RdRPs identified through the phylogenetic results (Figure 6), suggesting a better adaptation of the mite RdRPs than the putative viral donor relatives. For comparison, we included the RdRP sequence of Clerodendrum chlorotic spot virus (ClCSV, *Dichorhavirus clerodendri*), and CiLV-C, both speculated to replicate in the mite, which also showed lower indexes of 0.66 and 0.57, respectively (Figure 6). However, we also identified four transcripts with ORFs of complete viral RdRPs in the additional metagenomic libraries of *Brevipalpus yothersi* as part of the virome analysis [66], resulting in indexes ranging from 0.70 to 0.75, which were higher values than those of the mite RdRPs integrated into the genome (Figure 6), suggesting that some viruses identified in the mite transcriptome may already exhibit high CAI values. Furthermore, three of those transcripts were classified as mite/eukaryotic by the ML model (Table S5B). Interestingly, we found a transcript from the *B. obovatus* transcriptome with no detectable match in its reference genome that showed a striking percentage of identity at the protein level with the genomic RdRPs of *B. yothersi*. It might represent the closest relative to the viral donor of the HGT event, although such transcript could not be confirmed in the *B. yothersi* transcriptome.

## 4. Discussion

Two RdRPs have been identified in the genome of *Brevipalpus yothersi,* likely of viral origin. This finding was supported by protein sequence similarity, domain analysis, 3D structure and phylogenetic inference. Both RdRPs showed kinship with partitivirids. Several dsRNA viruses have been reported to participate in HGT events in eukaryotes [25,84,85], and partitiviruses in particular. Metagenomic studies indicate that, besides fungi, plants, and protozoans, these viruses are also associated with arthropods [86]. They are almost exclusively vertically transmitted, and reverse transcriptase activity has been experimentally demonstrated for the RdRPs of this viral family [26,87], phenomena that could enhance integration events [25,86]. The confirmation of expression of both RdRPs by RNAseq and molecular assays, presence of complete ORFs, complete domains and conserved active sites on the deduced protein sequences suggest functionality and selective pressure to retain protein-coding capacity. Moreover, deregulation across developmental stages suggests precise transcriptional regulation, pointing toward a functional role in mite biology. Thus, the first relevant question revolves around the role that those RdRPs might have in the mite. The domain identified in the mite RdRPs (PF00680) is present in replicases of both single- and double-stranded RNA viruses [20,88]. In dsRNA viruses, the RdRP uses the viral genome as a template to transcribe +RNA into mRNAs, and in +ssRNA viruses, it creates a negative-sense strand intermediate to replicate the virus. Although replication complexes in vivo require cis-acting cofactors and a very specific molecular context, this raises the possibility that either of the mite RdRPs could be co-opted by any mite-infecting virus and contribute to its replication, at least partially. It is well known that many host factors are recruited by viruses for propagation, and some studies have reported exceptional cases of molecular compatibility between viral sequences and even eukaryotic replicases, for example DNA-dependent RNA pol II and nuclear-replicating viroids [89,90], or hepatitis delta virus (HDV) [91]. Replication of CiLV-C in *Brevipalpus yothersi* has been controversial for the last few years. The detection of anti-genomic viral RNA was considered evidence of replication [92], but viroplasms have never been observed by electron microscopy and virions have never been detected within mite cells, being only found in the intercellular spaces [93]. However, this cilevirus is able to establish a persistent circulative transmission [94], and under high-stringency, anti-genomic RNAs were present at low but stable levels over the mite life cycle, suggesting that CiLV-C replicates at a very low rate [95]. Therefore, even in the absence of complete compatibility of the CiLV-C machinery with the host, the viral nature of the mite RdRPs might facilitate exceptional interactions and provide a synergistic relationship with the virus to be further investigated, although this hypothesis remains largely speculative.

Conversely, the link between EVEs and antiviral immunity is supported by many more studies. A hypothetical recruitment of either mite RdRP against CiLV-C would enable an antagonistic role, whether through participating in the RNA interference response, viral protein sequestration, or any other mechanism that might eventually help control viral accumulation. EVEs on eukaryotic genomes, for example, are often a source of small RNAs, particularly piRNAs in arthropods [96], and may eventually participate in immunity against cognate viruses, including non-retroviral integrations [97]. How persistence is established in vector–virus interactions without a fitness cost to the vector is a longstanding question that remains poorly understood, in part due to the limited efforts to study viral intermediates in pathosystems. In certain arthropods, some endogenous elements control viral replication to avoid host damage, allowing the persistent stage to be established [98,99,100], many of them being related to the silencing machinery at the RNA level. In our work, we confirmed that both RdRPs were down-regulated in CiLV-C-viruliferous adult mites, taking into account the necessary caveats, since viruliferous and aviruliferous samples were reared on different substrates due to methodological limitations. A functional genomics approach, such as silencing the mite RdRPs by RNAi, will be essential to answer all these questions, and our research is currently moving in this direction.

Generally speaking, arthropods play a prominent role as vectors of viruses, and their virome content is reportedly abundant and complex [101], providing opportunities for genetic recombination and exchange. In the virome of *Tetranychus urticae*, for example, several viral families, new viruses or their isolates have been identified across different mite populations [102]. In the case of *Brevipalpus*, preliminary results also show high complexity and the presence of disparate viral families [66], which provides the same aforementioned opportunities for potential antagonistic or synergistic interactions between the mite genomic RdRPs and those viruses for further research, other than CiLV-C. It should be noted that despite the high number of reported *Brevipalpus*-transmitted viruses, virtually all of them are non-pathogenic to these mites. Just one study suggested an overall reduction in reproductive potential for mites feeding on CiLV-C-infected oranges, although it is not clear whether this is attributable to the nutritional value of infected plants, rather than to a direct effect of CiLV-C in the mite [103]. This contrasts strongly with insects, where viral infections with a fitness cost for the host, or even lethal viruses, are described [104]. The extent to which the virus-derived mite RdRPs identified in our work (seemingly linked to the genus) contribute to improved immunity in *Brevipalpus* mites poses another avenue for investigation.

Far beyond virus–host interactions, additional functions could have been gained in the mite as a consequence of the HGT event related to the biological processes in which canonical eukaryotic RdRPs are involved, such as control of transposon proliferation, RNA amplification, or non-canonical RNA synthesis. This idea is especially appealing since the genomes of most Acari species encode eukaryotic RdRPs [17], except for *B. yothersi*. Indeed, the non-polymerase roles attributed to viral RdRPs add an additional layer of potential molecular innovations for the mite as exapted RdRP genes, assuming that a real endogenization event occurred. The presence of several orthologs in different *Brevipalpus* species, supported by transcriptome data and a phylogenetic topology largely congruent with the *Brevipalpus* phylogeny, argues in favor of a true viral gene domestication by the genus, as does the expression pattern observed across the four developmental stages in *B. yothersi*. Understanding these functional roles might be paramount for other goals. For example, RNAi pesticides constitute an effective and environmentally friendly alternative to conventional chemical acaricides [105]. To overcome off-target effects, highly specific gene content of the pest is selected during target design. If a true biological role of those virus-derived RdRPs were confirmed (whether immunity-related or endogenous silencing), they might be excellent candidates for species-specific targets during RNAi pesticide design, due to their low sequence similarity to other eukaryotic genes, providing an interesting opportunity to be explored for pathogen control in *Brevipalpus*.

We also aimed to characterize the mite RdRPs at the nucleotide sequence level by analyzing base compositional biases using k-mer profiles. In humans, for example, k-mer occurrence in ancient viral RNA sequences remains similar to that of extant RNA viral sequences and can be differentiated from that of other human genome sequences [24]. Unexpectedly, both mite RdRPs were classified as eukaryotic using an ML model when compared with mite and viral genes, contrasting with their robust classification as viral at the protein sequence level. One explanation is that the mite RdRPs may have undergone gene amelioration (e.g., codon usage adaptation) after integration. However, similar CAI values were also retrieved for some of the uncharacterized RdRPs identified in the *Brevipalpus* virome, some even exceeding the average for mite genes in one case, and most of them also lacking statistical power to be classified as viral by the ML model, therefore mimicking the k-mer profiles of mite genes (Table S5). In this regard, k-mer occurrence in viral genomes is believed to be shaped by several selective constraints, including codon usage bias, which has been proposed to be modulated in different ways depending on the host–pathogen interaction [106,107,108,109]. Certainly, codon usage adaptation is not the only parameter determining virus–host compatibility, and ClCSV is a good example, known to replicate in *B. yothersi* but with a very low CAI value. Thus, the high CAI values and k-mer resemblance of those RdRPs from the mite virome are suggestive of a tight relationship that could bring biological features far beyond mere translation efficiency, such as facilitating integration events. This hypothesis is in line with some studies in bacteria that show how the success of HGT events is impacted by codon usage, not due to gene amelioration (i.e., post-transfer adaptation), but rather due to the donor sequence already possessing a codon bias similar to that of the recipient organism [77,78]. In any case, without knowing the exact viral donor we cannot ascertain which factor contributed the most, nor the extent to which codon adaptation is the driving force, but our results raise the possibility that the genomic RdRPs in *B. yothersi* were derived from a viral donor already adapted to the host, or at least from a donor mimicking the k-mer content of the mite genome by any mechanism. Among them, the viral RdRP transcripts identified in *B. obovatus* and *B. chilensis* (brob.Viral and brchi.Viral), although uncharacterized and not confirmed by sequencing data in *B. yothersi*, represent the best candidates as the closest relatives to this donor that we were able to identify.

## Figures and Tables

**Table 1:** Protein sequences used for 3D structure analysis.

Number of Modeled Residues	Protein Sequence Region	Pfam ID	Source	Protein Accession	Protein ID	Organism
383	29–411	PF00680	AlphaFold	DN636_c1_g1_i8	RdRP1	*B. yothersi*
369	38–406	PF00680	AlphaFold	DN100_c0_g1_i88	RdRP2	
436	2057–2492	PF00978	AlphaFold	DQ352194	CiLV-C	Citrus leprosis virus C
1032	38–1069	PF00946	Cryo-EM	5a22	VSIV	Vesicular stomatitis Indiana virus
441	A 38–478	PF00680	X-Ray	1khv	RHDV	Rabbit hemorrhagic disease virus
290	83–372	PF00680	AlphaFold	APG78281	HPLV-42	Hubei partiti-like virus 42
209	37–245	PF00680	AlphaFold	XP_074603960	Bo_1	*B. ovobatus*
364	38–401	PF00680	AlphaFold	XP_074602295	Bo_2	
487	31–420	PF00680	AlphaFold	XP_074604054	Bo_3	
476	6–398	PF00680	AlphaFold	XP_074604223	Bo_4	
619	464–1082	PF05183	AlphaFold	XP_015794858	Tu_1	*T. urticae*
620	461–1080	PF05183	AlphaFold	XP_015794860	Tu_2	
604	424–1027	PF05183	AlphaFold	XP_015794862	Tu_3	
532	493–1024	PF05183	AlphaFold	RWS09175	Dt_1	*D. tinctorium*

## Data Availability

The genome and transcriptome data presented in the study are openly available in GenBank (assembly GCA_003956705.1, Bioproject PRJNA490612). The raw virome data presented in this article are not readily available, as they are part of an ongoing study [66]. Only viral FASTA sequences identified in our study are available upon request to the corresponding author. Requests to access the raw data should be directed to Daniel Carrillo.

## Supplementary Materials

The following supporting information can be downloaded at https://www.mdpi.com/article/10.3390/v18080892/s1: Figure S1. Phylogenetic tree of viral RNA-dependent RNA polymerases. Unrooted phylogenetic tree of viral RdRPs along with RdRP1 and RdRP2 from *Brevipalpus yothersi*. Different colors are used to represent different viral taxa. Order (O); phylum (P). *B. yothersi* protein IDs are marked with a star. Figure S2. All-vs-all matrix comparison of protein sequences of RdRPs from *Brevipalpus yothersi* and *Brevipalpus obovatus*. Identity (green) and positives (orange) derived from BLAST analysis. Protein ID description is provided in Table 1. Figure S3. Analysis of *Brevipalpus* orthology. (A) Phylogenetic tree of RdRPs from different species of *Brevipalpus*, Hubei partiti-like virus 42 was used as outgroup to root the tree. Species are colored based on the taxonomy grouping proposed by [67]. (B) Species and assembled transcript IDs used to build the phylogenetic tree. Figure S4. RMSD and number of aligned residues from a pairwise structure alignment of selected RdRPs. Dual triangular matrix representing both RMSD in the upper triangular matrix (blue) and aligned residues in the lower triangular matrix (red). For information about the proteins represented, see Table 1; Table S1. Sequence of transcripts from *Brevipalpus yothersi* with similarity to RdRPs. (A) Predicted amino acid sequence, (B) Selected transcripts as full-length representatives of the mite endogenous RdRPs. Table S2. Primers for qPCR analysis of RdRPs. Table S3. Putative orthologs of RdRP1 and RdRP2 from *Brevipalpus yothersi* identified in the transcriptome of *Brevipalpus* ssp. Table S4. FoldSeek analysis. Results of FoldSeek when RdRP1 and RdRP2 from the mite were used as queries against the databases AlphaFold/Proteome, AlphaFold/Swiss-Prot, AlphaFold/UniProt50, PDB100, BFMD, BFVD, CATH50, GMGCL, Mgnify-ESM30 for the identification of 3D structural similarities. Table S5. Machine-learning model of DeepVirFinder. Nucleotide sequences used by the trained ML model of DVF. (A) Sequence IDs from public repositories of viral or eukaryotic origin used to test the performance of the model, including the genomic RdRPs from *B. yothersi*, RdRPs from other mite species, RdRPs of viruses derived from the phylogenetic or protein 3D analyses of our study, genomic RNA from several isolates of CiLV-C, and several partitiviruses or durnaviruses. (B) RdRPs from viruses identified in the virome of *B. yothersi* classified by the DVF ML model as viral or eukaryotic, and calculated Codon Adaptation Index (CAI) to the codon usage of *B. yothersi*.; Text S1. Protein sequence details of active sites identified by RPS-BLAST.
