# Supplementary material for: Identification of Partitivirus-like RdRPs in the Brevipalpus yothersi Genome Supports Viral-to-Arthropod Horizontal Gene Transfer
Source: Viruses. 2026 Aug 13;18(8):892. doi: 10.3390/v18080892 (PMC13517406; doi:10.3390/v18080892)
Supplement: Supplementary file 1 [file viruses-18-00892-s001.zip › Supplementary Text S1.pdf]

Text S1. Protein sequence details of active sites identified by RPS-BLAST.

The RdRp-scan resource has a collection of motifs A, B and C from different taxa of viruses which are broadly conserved in RdRps and belong to the palm subdomain, responsible for the catalysis. We identified that the first nucleotide binding site (in green) of *B. yothersi* in Figure 1 (DFKxFD) is the A motif of *Durnavirales*, the second (SG) is part of B motif (SGSxFTxLxG) and the third (DD) is part of C motif (GDD). The fourth one is a LG motif identified and classified as a generic active site by CDD, which may be part of the D motif of the RdRPs. The residues flagged as "x" are those for which the two proteins of the mite do not match.

Brevip*alpus yothersi*:

RdRP1:

RPS-BLAST output:

1 Query_1 Superfamily 477363 31 412 4.09746e-22 98.6365 cl40470 ps-ssRNAv_RdRp-like - -

SITES

1 Query_1 Generic active site D233,F234,K235,T236,F237,D238,S306,G307,D342,D343,L385,G386 13 12 238185

1 Query_1 Generic nucleic acid binding site G307 1 1 238185

1 Query_1 Generic NTP binding site D233,F234,K235,T236,F237,D238,S306,D342 8 8 238185

Protein sequence:

MGHIQKKPPHLSMKCEFIGEAEPFKMTFAWDTSPSSLLPEYAKYARHLWRSPCSEDEYFNDIL
DYSNTVGQPQNPFLSYAIFLANEAFRLPKKVPMIKLEDVFGRTDLHIWKSSPGLPWKEKGYRTK
SDIRGNPAAVDAIIDFWNDILNGKDREPDDTMVVNKRHITDRGNHKIRAIMCYPATINFGEVIFTT
PLTDEYVKGGSPIAFGYETLRGGAMKISRECIAEWYTASDFKTFDKSVPVFLLQAAFNILKSNID
FNAYQNTNELPNVHRMEMMFDYLRNYFIHTPFRTFDGYRYRKHGGIASGSGFTTLIGSIVNYILI
QWASLDQNGVPRYIKVFGDDSICGHSYKFDIEKANRLFTQIRMSLNLNKSKVTKSLAELSFLGF
QINYGYPKKEFDRWMASLIYPETNDKDWDTVASRAKSLLYCCCGFNLEFDKICREIIGRRDFEL
KESKRTHYIRMLGMDLDDLKGPVPSPEQLRRRLTEV

- The DFKTFD sequence is the A motif of the palm domain from *Durnavirales*, with a T in the place of a S.
- The purple sequence is possibly the B motif from *Durnavirales*, SGSYFTQLID, but only the first two amino acids were identified by RPS-BLAST.
- The DD sequence is part of a GDD motif, the C motif of the palm domain from a lot of viruses

- The LG sequence may be part of the D motif of the RdRPs.

RdRP2:

RPS-BLAST output:

1 Query_1 Superfamily 477363 37 408 8.41999e-29 118.282 cl40470 ps-ssRNAv_RdRp-like - -

SITES

1 Query_1 Generic active site D228,F229,K230,S231,F232,D233,S301,G302,D337,D338,L380,G381 13 12 238185

1 Query_1 Generic nucleic acid binding site G302 1 1 238185

1 Query_1 Generic NTP binding site D228,F229,K230,S231,F232,D233,S301,D337 8 8 238185

Protein sequence:

MSGECEYLGKWDGFRMTYIKDTEPTKFLRQYHVHAREYWRSPASEADLRKDVLAYGNTVKRP
NDRSLSYAIHLANEAFKLPQKVKMLALNDVFAQDDLGIWDKSPGLPWIKKNFKSKNDIRRDSEA
TKQVINFWEKVKKGQERLPDDTCAFVRRHIVKREKIKVRAILGYPATITFGEVVFAHQLTQTYLD
LRLTAAASPLAYGYETQLGGAMRIFQECYADWYVATDFKSFDKTVPEFLIEAAFNILMSNIDFEH
YHESDIVPDAREMVLMFRYLVKYFIYTPIRFSNGERYRKKGGIASGSMFTQLVGSIVNFILIHWAS
LRQKGRPRYIKVFGDDSICGYHYKFNLATADDLFKSIRMSLNLDKSSVTNHLADVKFLGYKINNG
LPSRDFQQWMASLLYPERPDTHWKYVATRALSLIYCNGGIDERFDKICRTIIRRHKFEIVLSKHQ
KNHLKMIGIEPETITPDPPERSELIRNLTHIDMNPRDDQVEN

- The DFKSFD sequence is the A motif of the palm domain from *Durnavirales*.
- The purple sequence is possibly the B motif from *Durnavirales*, SGSYFTQLID, but only the first two amino acids were identified by RPS-BLAST.
- The DD sequence is part of a GDD motif, the C motif of the palm domain from a lot of viruses.
- The LG sequence may be part of the D motif of the RdRPs.

White clover cryptic virus 1 (WCCV-1):

UniProtKB/Swiss-Prot accession AY705784:

RPS-BLAST output:

1 Query_1 Superfamily 477363 243 518 3.73599e-12 68.5909 cl40470 ps-ssRNAv_RdRp-like N -

SITES

1 Query_1 Generic active site D314,W315,S316,R317,F318,D319,S397,G398,Q433,D435,D436,L486,S487 13 13 238185

1 Query_1 Generic nucleic acid binding site G398 1 1 238185

1 Query_1 Generic NTP binding site D314,W315,S316,R317,F318,D319,S397,D435 8 8 238185

Protein sequence:

MDYLITAFNRITHWFLTPTNLEYIGSYSLPPGLLRVNDVAVANHKATLDRSFDKYLYEHEINLITK
EYRRSPIDEDSILEDFFSGDLPYFEIPFDEHVERGLECMAAAFRPPRPCRPAHILDVKHGYPYK
WNVNAEPPFSTDEYFLSQRKTFGEFIRMHEYEHIDKEDFFRRHPNIESHDFLRTVVPPKFGFLK
SMIFSWTRRWHHIIKSGFQDSTDLEQTGYFFNRFIFPMLLHTKTAIVKKNDPNKMRTIWGASKP
WIIAETMFYWEYLAWIKHNPGATPMLWGYETFTGGWFRLNHELFCGLIQRSFLTLDWSRFDKR
AYFPLLRRILYTVKTFLTFEEGYVPTHAAPTHPQWSQENIDRLERLWLWTLENLFEAPIILPDGR
MYRRHFAGIPSGLFITQLLDSWYNYTMLATILSALHFDPLHCIIKVQGDDSILRLTTLIPVDQHTNF
MDHIVRLADTYFNSIVNVKKSEVRNSLNGCEVLSYRNHNGLPHRDEITMLAQFYHTKARDPTPE
ITMAQAIGFAYASCANHNRVLWVLEDVYNYYRDLGYRPNRAGLTLTFGDSPDLTMPEMPLDHF
PTKSEIRRYHTETHYQNEAQNARTWPRTLFINAPGE

- The DWSRFD sequence is the A motif of the palm domain from *Durnavirales*, with a T in the place of a S.
- The purple sequence is the B motif from *Durnavirales*, SGLFITQLLD, but only the first two amino acids were identified by RPS-BLAST.
- The DD sequence is part of a GDD motif, the C motif of the palm domain from a lot of viruses.
- The LS sequence may be part of the D motif of the RdRPs.
