# Supplementary material for: Identification of Partitivirus-like RdRPs in the Brevipalpus yothersi Genome Supports Viral-to-Arthropod Horizontal Gene Transfer
Source: Viruses. 2026 Aug 13;18(8):892. doi: 10.3390/v18080892 (PMC13517406; doi:10.3390/v18080892)
Supplement: Supplementary file 1 [file viruses-18-00892-s001.zip › Supplementary Figure S1.pdf]

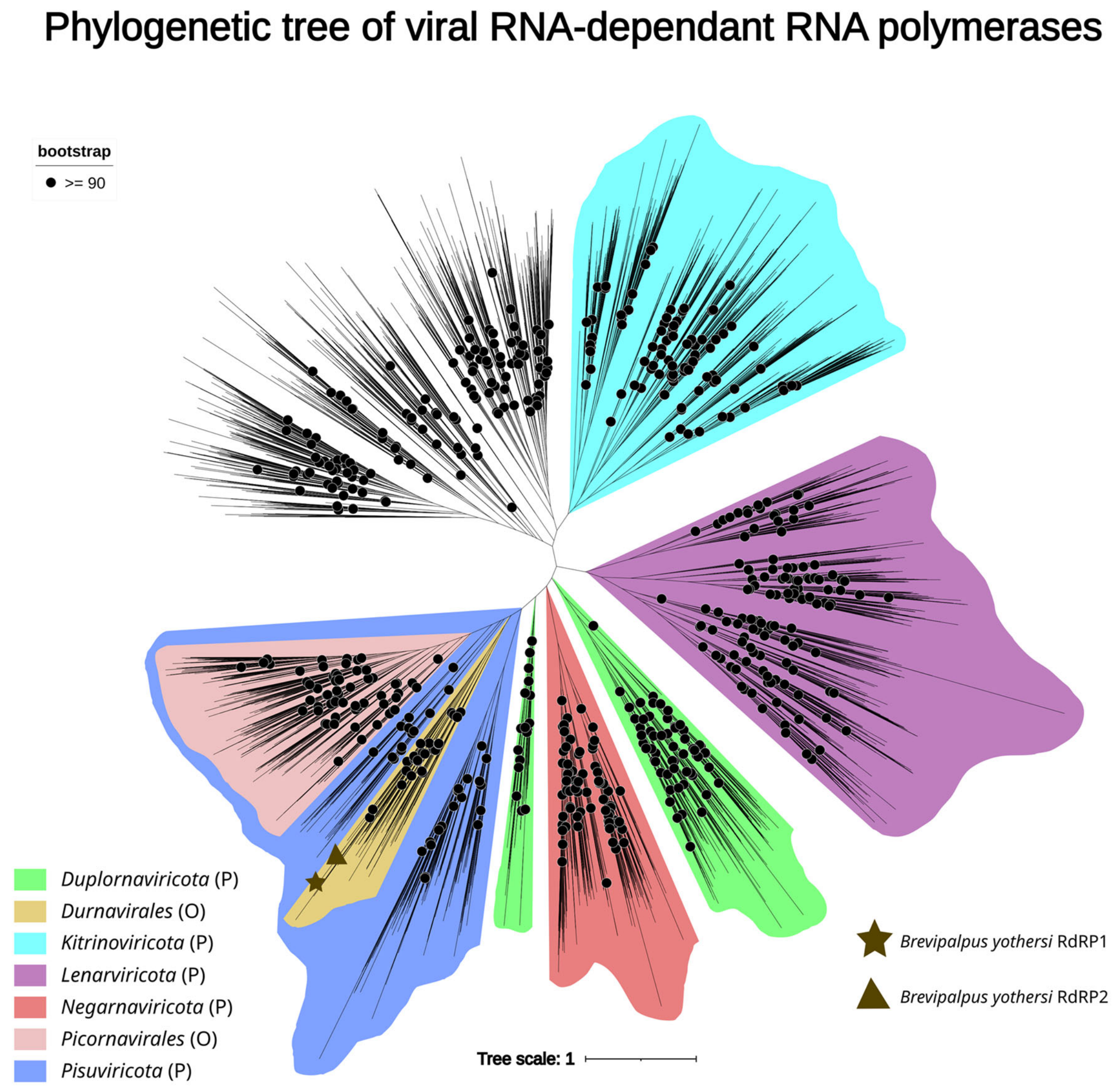

Figure S1. Phylogenetic tree of viral RNA-dependent RNA polymerases. Unrooted phylogenetic tree of viral RdRPs along with RdRP1 and RdRP2 from *Brevipalpus yothersi*. Different colors are used to represent different viral taxa, order (O), phylum (P). *B. yothersi*'s proteins are marked with a star.
