# Supplementary material for: Identification of Partitivirus-like RdRPs in the Brevipalpus yothersi Genome Supports Viral-to-Arthropod Horizontal Gene Transfer
Source: Viruses. 2026 Aug 13;18(8):892. doi: 10.3390/v18080892 (PMC13517406; doi:10.3390/v18080892)
Supplement: Supplementary file 1 [file viruses-18-00892-s001.zip › Supplementary Figure S2.pdf]

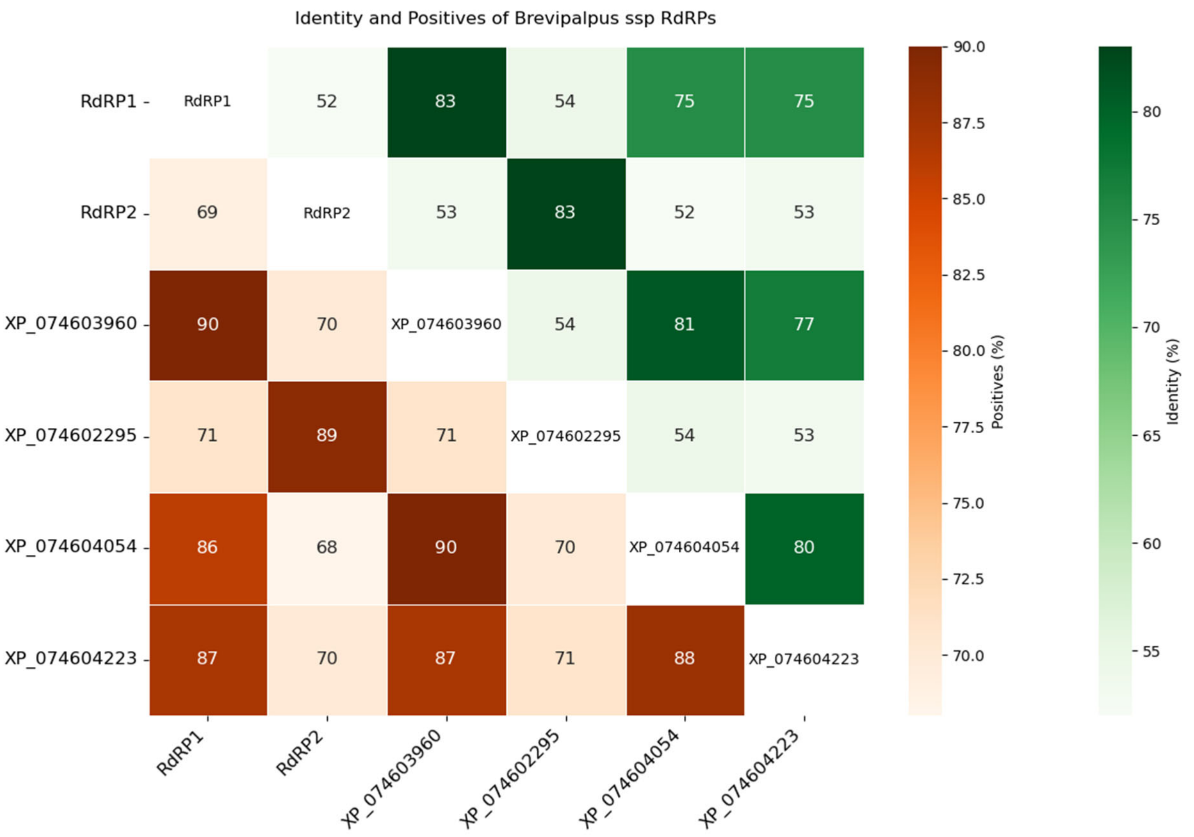

Figure S2. All-vs-all matrix comparison of protein sequences of RdRPs from *Brevipalpus yothersi* and *Brevipalpus obovatus*. Identity (green) and positives (orange) derived from BLAST analysis. Protein ID description is provided in Table 1.
