# Supplementary material for: Identification of Partitivirus-like RdRPs in the Brevipalpus yothersi Genome Supports Viral-to-Arthropod Horizontal Gene Transfer
Source: Viruses. 2026 Aug 13;18(8):892. doi: 10.3390/v18080892 (PMC13517406; doi:10.3390/v18080892)
Supplement: Supplementary file 1 [file viruses-18-00892-s001.zip › Supplementary Figure S3.pdf]

Analysis of Brevipalpus orthology

A

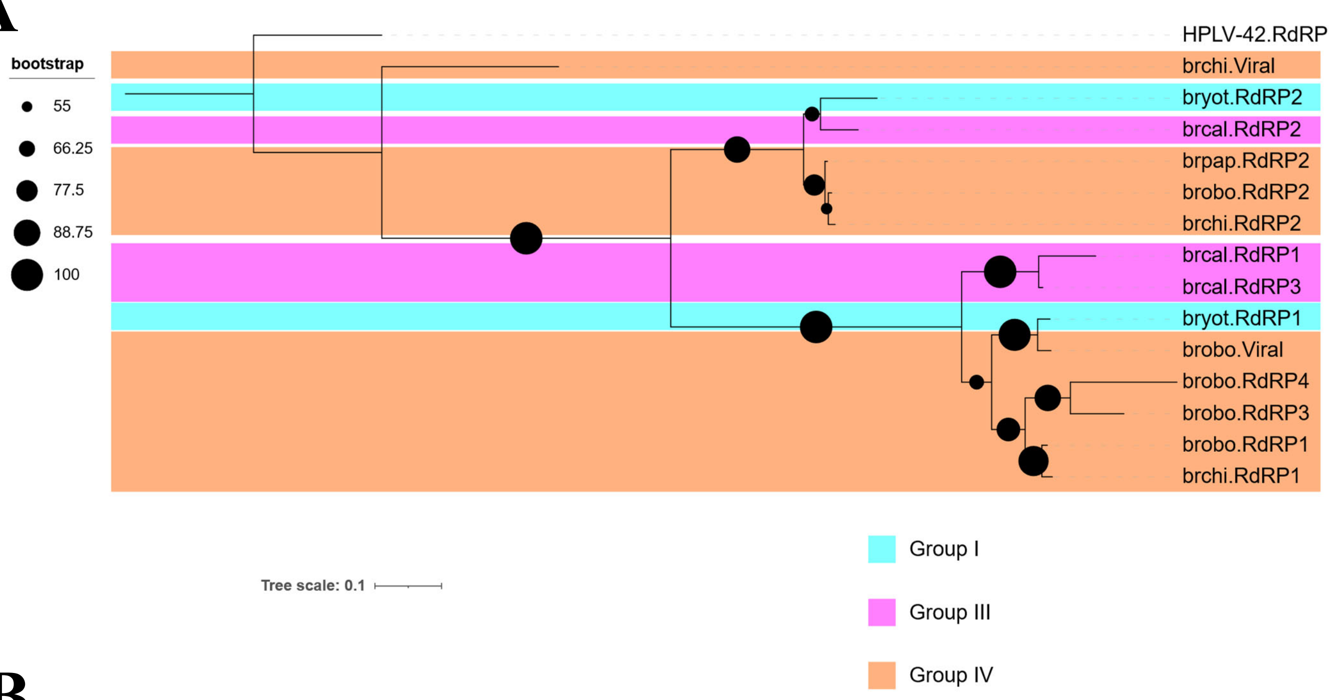

B

SPECIES	TRINITY ID / ACCESSION	NAME IN THE TREE
B. chilensis	TRINITY_DN1555_c0_g1_i3	brchi.RdRP2
	TRINITY_DN43408_c0_g1_i1	brchi.Viral
	TRINITY_DN5303_c0_g1_i1	brchi.RdRP1
B. yothersi	TRINITY_DN636_c1_g1_i8	bryot.RdRP1
	TRINITY_DN100_c0_g1_i88	bryot.RdRP2
B. papayensis	TRINITY_DN40567_c0_g1_i1	brpap.RdRP2
B. californicus	TRINITY_DN5110_c0_g4_i1	brcal.RdRP2
	TRINITY_DN7072_c0_g1_i14	brcal.RdRP1
	TRINITY_DN7072_c0_g1_i15	brcal.RdRP3
B. obovatus	TRINITY_DN18007_c0_g1_i1	brobo.Viral
	XP_074603960	brobo.RdRP1
	XP_074602295	brobo.RdRP2
	XP_074604054	brobo.RdRP3
	XP_074604223	brobo.RdRP4

Hubei partiti-like virus 42	APG78281	HPLV-42

Figure S3. Analysis of *Brevipalpus* orthology. (A) Phylogenetic tree of RdRPs from different species of *Brevipalpus*, Hubei partiti-like virus 42 was used as outgroup to root the tree. Species are colored based on the taxonomy grouping proposed by Mendonça et al, 2025 **(B)** Species and assembled transcript IDs used to build the Phylogenetic tree.
