# Supplementary material for: Identification of Partitivirus-like RdRPs in the Brevipalpus yothersi Genome Supports Viral-to-Arthropod Horizontal Gene Transfer
Source: Viruses. 2026 Aug 13;18(8):892. doi: 10.3390/v18080892 (PMC13517406; doi:10.3390/v18080892)
Supplement: Supplementary file 1 [file viruses-18-00892-s001.zip › Supplementary Figure S4.pdf]

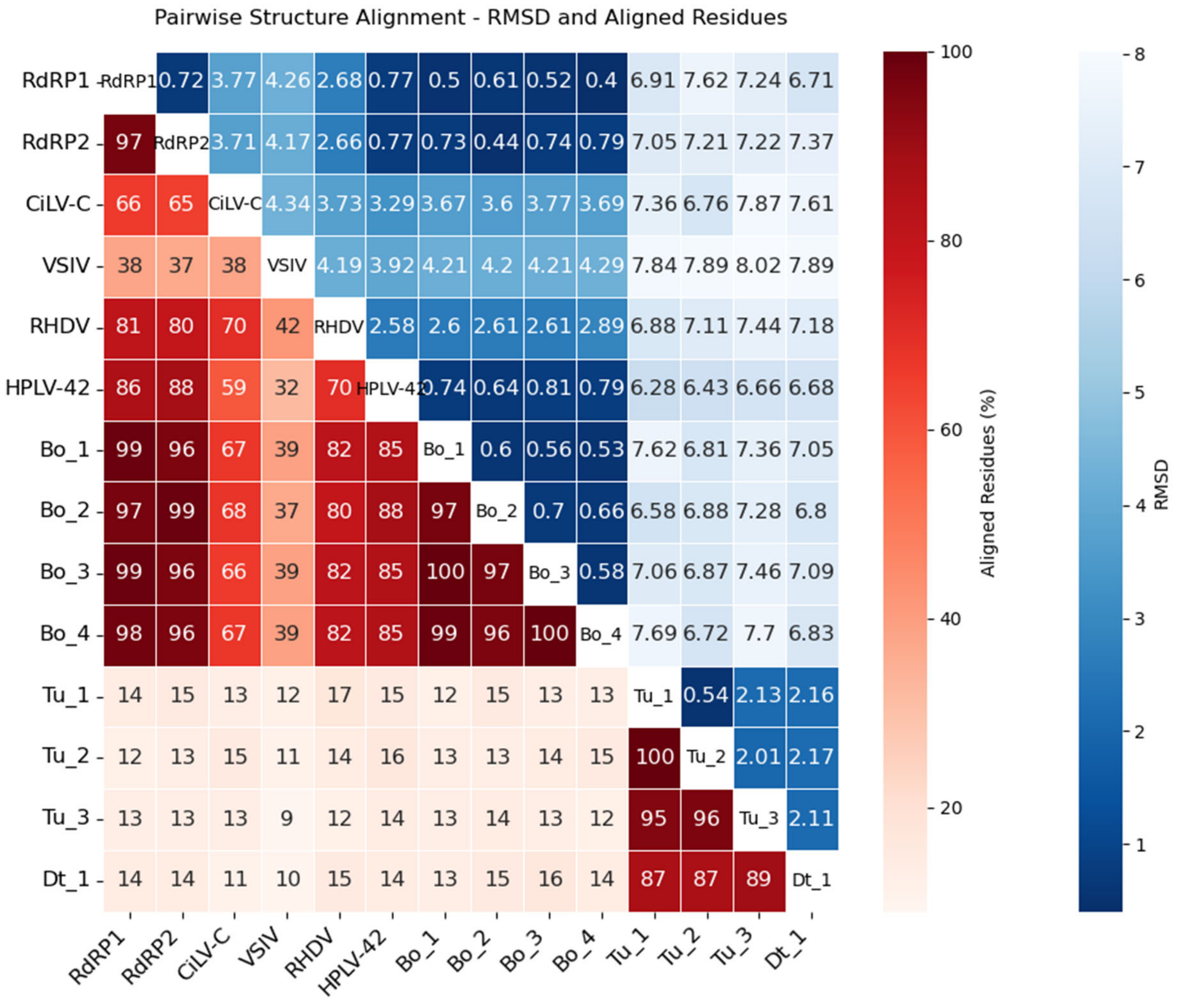


**Figure S4. RMSD and number of aligned residues from a pairwise structure alignment of selected RdRPs.** Dual triangular matrix representing RMSD in the upper half (blue) and aligned residues in the lower half (red). For information about the proteins represented, see Table 1.
