# Supplementary material for: Identification of Partitivirus-like RdRPs in the Brevipalpus yothersi Genome Supports Viral-to-Arthropod Horizontal Gene Transfer
Source: Viruses. 2026 Aug 13;18(8):892. doi: 10.3390/v18080892 (PMC13517406; doi:10.3390/v18080892)
Supplement: Supplementary file 1 [file viruses-18-00892-s001.zip › Supplementary Table S1.pdf]

Table S1. Sequence of transcripts from *Brevipalpus yothersi* with similarity to RdRPs.

A. Predicted amino acid sequence

InterProScan-Trinity contig	Aminoacid sequence of predicted ORF
TRINITY_DN100_c0_g1_i120.p1<br>TRINITY_DN100_c0_g1_i153.p3<br>TRINITY_DN100_c0_g1_i157.p3<br>TRINITY_DN100_c0_g1_i21.p3<br>TRINITY_DN100_c0_g1_i39.p3<br>TRINITY_DN100_c0_g1_i50.p3<br>TRINITY_DN100_c0_g1_i56.p3<br>TRINITY_DN100_c0_g1_i59.p3<br>TRINITY_DN100_c0_g1_i84.p3<br>TRINITY_DN100_c0_g1_i88.p1	MSGECEYLGKWDGFRMTYIKDTEPTKFLRQYHVHAREYWRSPASEADLRKDVLAYGNTVKRPNDRSLSYAIHLANEAFKLPQKVKMLALNDVFAQDDLGIWDKSPGLPWIKKNFKSKNDIRRDSEATKQVINFWEKVKKGQERLPDDTCAFVRRHIVKREKIKVRAILGYPATITFGEVVFAHQLTQTYLDLRLTAAASPLAYGYETQLGGAMRIFQECYADWYVATDFKSFDKTVPEFLIEAAFNILMSNIDFEHYHESDIVPDAREMVLMFRYLVKYFIYTPIRFSNGERYRKKGGIASGSMFTQLVGSIVNFILIHWASLRQKGRPRYIKVFGDDSICGYHYKFNLATADDLFKSIRMSLNLDKSSVTNHLADVKFLGYKINNGLPSRDFQQWMASLLYPERPDTHWKYVATRALSLIYCNGGIDERFDKICRTIIRRHKFEIVLSKHQKNHLKMIGIEPETITPDPPERSELIRNLTHIDMNPRDDQVEN - 494 aa
TRINITY_DN2153_c0_g2_i1.p1	MSTHN…LPVIN - 2512 aa
TRINITY_DN2153_c0_g2_i4.p1	SRLGQ…DIKKI - 1676 aa
TRINITY_DN2153_c0_g2_i4.p3	MALCARNFDLSKLKLAVFSGDDSLLVGEKRDLQCDSQNFSDLFNLDVKFFPNFKYYHFCSKFLIAVEDRWYFIPDPVKLCVRLARLDLVNWAHIEEYRISLKDTTKYYCDDGIARELSRAVCDRYLVVVDPVEVLRVVCSLVSCKDEFRLLFEEPIACLPEGNLLPVIN - 169 aa
TRINITY_DN636_c1_g1_i11.p1<br>TRINITY_DN636_c1_g1_i3.p1<br>TRINITY_DN636_c1_g1_i6.p1<br>TRINITY_DN636_c1_g1_i7.p2	MQYSWPMKLSDYPKKVPMIKLE..RLTEV - 409 aa*
TRINITY_DN636_c1_g1_i4.p1<br>TRINITY_DN636_c1_g1_i8.p1	MGHIQKKPPHLSMKCEFIGEAEPFKMTFAWDTSPSSLLPEYAKYARHLWRSPCSEDEYFNDILDYSNTVGQPQNPFLSYAIFLANEAFRLPKKVPMIKLEDVFGRTDLHIWKSSPGLPWKEKGYRTKSDIRGNPAAVDAIIDFWNDILNGKDREPDDTMVVNKRHITDRGNHKIRAIMCYPATINFGEVIFTTPLTDEYVKGGSPIAFGYETLRGGAMKISRECIAEWYTASDFKTFDKSVPVFLLQAAFNILKSNIDFNAYQNTNELPNVHRMEMMFDYLRNYFIHTPFRTFDGYRYRKHGGIASGSGFTTLIGSIVNYILIQWASLDQNGVPRYIKVFGDDSICGHSYKFDIEKANRLFTQIRMSLNLNKSKVTKSLAELSFLGFQINYGYPKKEFDRWMASLIYPETNDKDWDTVASRAKSLLYCCCGFNLEFDKICREIIGRRDFEL

	KESKRTHYIRMLGMDLDDLKGPVPSPEQLRRRLTEV - 487 aa*

*Amino acid composition differences based on transcript splicing variants, or assembly errors are highlighted in yellow.

B. Selected transcripts as full-length representatives of the mite endogenous RdRPs.

>bryot.RdRP1 DN636 cds length:1464
ATGGGACATATCCAGAAAAAGCCACCGCATTTGTCGATGAAATGTGAATTTATTGGTGAA
GCAGAGCCATTTAAAATGACATTCGCCTGGGATACTTCACCATCTTCCCTGTTACCAGAA
TATGCTAAATATGCTCGTCACTTGTGGAGAAGTCCATGTAGTGAGGATGAATATTTTAAT
GATATTTTGGATTACAGTAATACGGTAGGACAGCCACAAAATCCTTTTCTTTCGTATGCA
ATATTCTTGGCCAATGAAGCTTTCCGATTACCCAAAAAAGTGCCCATGATCAAATTGGAA
GATGTCTTTGGACGTACTGATTTACACATTTGGAAATCATCACCTGGTTTACCATGGAAG
GAGAAGGGATACCGAACCAAGAGTGATATTCGTGGGAATCCGGCGGCTGTGGATGCAATC
ATTGATTTTTGGAACGATATTCTTAATGGTAAAGATAGAGAACCAGATGACACGATGGTG
GTAAACAAAAGGCACATAACTGATCGAGGGAATCACAAAATCCGTGCCATAATGTGCTAT
CCAGCGACCATTAATTTTGGTGAAGTTATTTTCACCACTCCACTAACCGATGAATATGTA
AAAGGTGGATCTCCAATTGCTTTTGGCTATGAAACTCTTCGCGGAGGTGCGATGAAAATT
TCTCGTGAATGTATTGCTGAGTGGTATACTGCTAGTGATTTCAAGACATTTGACAAATCA
GTGCCTGTATTCCTGCTCCAAGCAGCCTTCAACATTCTTAAGTCGAACATCGATTTCAAT
GCCTACCAGAACACCAATGAACTTCCGAATGTGCATAGAATGGAGATGATGTTCGATTAT
CTTCGAAATTACTTTATTCACACGCCATTTCGTACCTTCGATGGATACAGATACAGGAAG
CATGGTGGCATAGCGAGTGGTTCTGGTTTCACCACACTTATTGGATCAATTGTTAATTAC
ATACTTATTCAATGGGCCAGTTTAGATCAAAATGGTGTTCCGAGATACATAAAAGTATTC
GGAGATGATTCAATTTGTGGCCACTCATACAAGTTTGACATCGAGAAAGCAAATAGACTA
TTCACCCAGATTCGGATGAGTCTGAATCTCAATAAATCCAAAGTTACGAAAAGTCTTGCC
GAACTTAGTTTCTTGGGCTTCCAAATTAATTATGGTTACCCTAAAAAGGAGTTTGATAGA
TGGATGGCTTCTCTGATCTATCCAGAGACAAATGATAAGGATTGGGACACTGTTGCTTCT
CGTGCCAAGAGTCTTCTGTATTGCTGCTGTGGTTTCAATCTGGAGTTCGATAAAATATGT
CGAGAAATTATTGGGAGAAGGGACTTTGAATTGAAAGAGTCGAAAAGAACACATTATATC
CGGATGCTTGGTATGGATCTAGATGACTTGAAGGGCCCCGTTCCTAGTCCGGAGCAACTT
CGCCGTCGGTTGACAGAGGTTTAG

>bryot.RdRP1 DN636 protein length:488
MGHIQKKPPHLSMKCEFIGEAEPFKMTFAWDTSPSSLLPEYAKYARHLWRSPCSEDEYFN
DILDYSNTVGQPQNPFLSYAIFLANEAFRLPKKVPMIKLEDVFGRTDLHIWKSSPGLPWK
EKGYRTKSDIRGNPAAVDAIIDFWNDILNGKDREPDDTMVVNKRHITDRGNHKIRAIMCY
PATINFGEVIFTTPLTDEYVKGGSPIAFGYETLRGGAMKISRECIAEWYTASDFKTFDKS
VPVFLLQAAFNILKSNIDFNAYQNTNELPNVHRMEMMFDYLRNYFIHTPFRTFDGYRYRK
HGGIASGSGFTTLIGSIVNYILIQWASLDQNGVPRYIKVFGDDSICGHSYKFDIEKANRL
FTQIRMSLNLNKSKVTKSLAELSFLGFQINYGYPKKEFDRWMASLIYPETNDKDWDTVAS
RAKSLLYCCCGFNLEFDKICREIIGRRDFELKESKRTHYIRMLGMDLDDLKGPVPSPEQL
RRRLTEV*

>bryot.RdRP2 DN100 cds length:1485
ATGTCAGGCGAATGTGAATATCTCGGCAAATGGGATGGATTTCGTATGACTTATATCAAA
GATACAGAGCCCACAAAATTTTTACGTCAGTATCATGTTCATGCTCGAGAATATTGGAGG
AGTCCAGCAAGTGAGGCTGATCTTCGCAAGGATGTTCTAGCTTATGGTAATACTGTGAAG
CGACCAAATGATCGCTCTCTGAGTTATGCGATTCATTTAGCAAATGAAGCTTTCAAGCTT
CCCCAAAAAGTGAAAATGCTCGCCTTGAATGATGTGTTCGCCCAAGATGACTTGGGAATA
TGGGACAAATCTCCTGGCTTGCCGTGGATTAAGAAAAATTTCAAATCTAAGAATGACATT
CGAAGAGATAGTGAAGCCACTAAGCAAGTTATTAACTTTTGGGAAAAAGTTAAGAAAGGA
CAGGAAAGGCTACCTGATGATACGTGTGCTTTCGTGCGAAGACATATTGTAAAACGTGAA
AAGATCAAGGTTCGAGCAATCCTAGGCTATCCTGCTACTATAACTTTTGGAGAGGTTGTG
TTTGCCCACCAATTGACTCAGACTTATTTAGATCTCAGACTCACTGCAGCTGCTTCACCA
CTTGCTTATGGTTACGAGACACAGTTGGGTGGTGCAATGAGAATATTTCAGGAATGTTAT
GCTGATTGGTACGTAGCAACCGATTTCAAATCATTTGACAAGACAGTTCCAGAGTTTCTG
ATCGAAGCAGCATTCAACATACTGATGTCCAACATTGACTTTGAGCACTATCATGAGAGC
GATATTGTTCCAGATGCTCGTGAAATGGTTCTTATGTTCAGGTATCTGGTGAAATACTTT
ATCTACACACCGATTCGTTTTAGTAATGGTGAGAGATATCGAAAGAAAGGAGGCATTGCC
AGCGGCTCGATGTTTACCCAATTGGTTGGCTCAATTGTGAATTTCATTCTGATTCATTGG
GCTAGTTTGAGGCAGAAAGGGCGCCCAAGATACATAAAAGTTTTTGGTGACGACTCAATT
TGTGGTTATCACTACAAATTTAATCTTGCTACTGCTGACGACCTATTTAAGTCGATACGC
ATGAGCCTTAATTTGGACAAATCTTCGGTCACTAATCACTTGGCTGATGTCAAATTCCTG
GGATATAAGATTAACAATGGATTGCCATCCCGTGACTTCCAGCAGTGGATGGCTTCTTTG

CTCTATCCTGAACGGCCTGATACACATTGGAAATACGTTGCAACGAGAGCCCTCAGTCTC
ATTTATTGTAATGGGGGTATAGACGAGCGATTCGATAAAATATGTCGTACTATAATTCGT
AGACATAAATTTGAGATTGTCTTGTCAAAGCATCAGAAAAATCACCTCAAAATGATCGGC
ATTGAACCAGAAACTATAACACCAGACCCACCAGAAAGATCTGAACTCATTCGCAATTTG
ACCCACATCGATATGAATCCAAGAGATGATCAAGTGGAAAACTGA

>bryot.RdRP2 DN100 protein length:495
MSGECEYLGKWDGFRMTYIKDTEPTKFLRQYHVHAREYWRSPASEADLRKDVLAYGNTVK
RPNDRSLSYAIHLANEAFKLPQKVKMLALNDVFAQDDLGIWDKSPGLPWIKKNFKSKNDI
RRDSEATKQVINFWEKVKKGQERLPDDTCAFVRRHIVKREKIKVRAILGYPATITFGEVV
FAHQLTQTYLDLRLTAAASPLAYGYETQLGGAMRIFQECYADWYVATDFKSFDKTVPEFL
IEAAFNILMSNIDFEHYHESDIVPDAREMVLMFRYLVKYFIYTPIRFSNGERYRKKGGIA
SGSMFTQLVGSIVNFILIHWASLRQKGRPRYIKVFGDDSICGYHYKFNLATADDLFKSIR
MSLNLDKSSVTNHLADVKFLGYKINNGLPSRDFQQWMASLLYPERPDTHWKYVATRALSL
IYCNGGIDERFDKICRTIIRRHKFEIVLSKHQKNHLKMIGIEPETITPDPPERSELIRNL
THIDMNPRDDQVEN*
