# Supplementary material for: Identification of Partitivirus-like RdRPs in the Brevipalpus yothersi Genome Supports Viral-to-Arthropod Horizontal Gene Transfer
Source: Viruses. 2026 Aug 13;18(8):892. doi: 10.3390/v18080892 (PMC13517406; doi:10.3390/v18080892)
Supplement: Supplementary file 1 [file viruses-18-00892-s001.zip › Supplementary Table S3.pdf]

Table S3. Putative orthologs of RdRP1 and RdRP2 from *Brevipalpus yothersi* identified in the transcriptome of *Brevipalpus ssp*.

Species	Trinity contig	RdRP1			RdRP2		
		len	%id	%pos	len	%id	%pos
B. chilensis	TRINITY_DN1555_c0_g1_i3	478	54,0	71,5	478	85,6	91,2
	TRINITY_DN43408_c0_g1_i1	465	44,5	61,9	451	50,6	67,6
	TRINITY_DN5303_c0_g1_i1	488	84,2	90,8	477	53,5	70,6
B. yothersi	TRINITY_DN1833_c0_g2_i1	488	99,8	99,8	477	53,5	70,9
	TRINITY_DN5005_c0_g3_i1	483	52,0	69,8	495	98,0	99,2
B. papayensis	TRINITY_DN40567_c0_g1_i1	289	58,8	74,4	289	88,6	94,5
B. californicus	TRINITY_DN5110_c0_g4_i1	434	53,9	74,7	446	84,8	91,9
	TRINITY_DN7072_c0_g1_i14	498	69,5	80,9			
	TRINITY_DN7072_c0_g1_i15				485	53,4	69,9
B. obovatus	TRINITY_DN18007_c0_g1_i1	488	95,1	97,7	477	53,7	70,9
	TRINITY_DN4744_c0_g1_i6	488	83,6	90,6	477	54,1	71,5
